# Rice-Husk Shredding as a Means of Increasing the Long-Term Mechanical Properties of Earthen Mixtures for 3D Printing

**DOI:** 10.3390/ma15030743

**Published:** 2022-01-19

**Authors:** Elena Ferretti, Massimo Moretti, Alberto Chiusoli, Lapo Naldoni, Francesco de Fabritiis, Massimo Visonà

**Affiliations:** 1 Department of Civil, Environmental and Materials Engineering—DICAM, Alma Mater Studiorum Università di Bologna, Viale del Risorgimento 2, 40136 Bologna, BO, Italy; 2 WASP s.r.l., Via Castelletto 104/106, 48024 Massa Lombarda, RA, Italy; massimo@3dwasp.com (M.M.); alberto@3dwasp.com (A.C.); lapo@3dwasp.com (L.N.); francesco@3dwasp.com (F.d.F.); massimo.visona@3dwasp.com (M.V.)

**Keywords:** earthen buildings, 3D printing, sustainability, biocomposites, aging, mechanical properties, lime carbonation, vulcanization

## Abstract

This paper is part of a study on earthen mixtures for the 3D printing of buildings. To meet the ever increasing environmental needs, the focus of the paper is on a particular type of biocomposite for the stabilization of earthen mixtures—the rice-husk–lime biocomposite—and on how to enhance its effect on the long-term mechanical properties of the hardened product. Assuming that the shredding of the vegetable fiber is precisely one of the possible ways to improve the mechanical properties, we compared the results of uniaxial compression tests performed on cubic specimens, made with both shredded and unaltered vegetable fiber, for three curing periods. The results show that the hardened earthen mixture is not a brittle material, in the strict sense, because it exhibits some peculiar behaviors that are anomalous for a brittle material. However, being a “designable” material, its properties can be varied with a certain flexibility in order to become as close as possible to the desired ones. One of the peculiar properties of the hardened earthen mixture deserves further investigation, rather than corrections. This is the vulcanization that occurs (in a completely natural way) over the long term, thanks to the mineralization of the vegetable fiber by the carbonation of the lime.

## 1. Introduction

The latest challenge in using raw earth for construction purposes is the additive manufacturing (3D printing) of entire housing modules. Of the three traditional earthen construction techniques—namely, adobe (compressed earth blocks), rammed earth (layers of damp earth compacted between formworks and left to dry once the formworks have been removed), and cob (Section 1.1 and Section 1.2)—cob is the most similar to the 3D printing of earthen buildings. In fact, both cob and 3D printing consist of laying a moist mixture of clay soil in superimposed layers to form a structure. Before adding a new layer on top of the previous one, both techniques require compliance with the setting times of the material. This requirement, known as the buildability criterion, leads to the fixing of the time taken to lay a layer in 3D printing equal to the setting time, in order to make the printing process continuous. One can actually see the 3D printing of earthen housing modules as the automated version of the manual cob technique.

Similar to cob (and unlike rammed earth), 3D printing does not require the use of formwork, which leads to numerous environmental benefits [1,2]. Additionally, both cob and 3D printing use a mixture consisting of subsoil (earth), water, and fibrous material. Both the preparation and the use of the fresh mixture take place on site (unlike adobe), which makes these techniques in-situ-based earthen construction techniques. The similarities between the two construction techniques allow for the transfer of vernacular knowledge and cob construction practices to additive manufacturing, resulting in the increased sustainability potential for applications in construction compared to concrete-based 3D-printing techniques [3].

Unlike cob, however, the printing process of earthen materials is very sensitive to the mixture, which is one of the main limiting factors in the development of the large-scale 3D printing of earthen buildings [4]. For example, the rheological behavior of the mixture must allow for a smooth extrusion through the 3D-printing system [4,5], despite the irregular particle size of the mixture and the addition of organic material. In principle, the use of finer portions of organic material facilitates the extrusion process.

According to [6], the basic criteria, which, if met, guarantee a successful 3D-printing process, are actually three: extrudability, buildability, and workability with time. The present work is part of the second line of research undertaken by the WASP (the World’s Advanced Saving Project) with the collaboration of the Department of Civil, Environmental and Materials Engineering of the University of Bologna (Italy), which deals with the mechanical characterization of the earthen mixes for 3D printing (Section 1.3). Since extrudability, buildability, and workability are topics of the WASP’s first line of research (Section 1.3)—from which the mixtures used in the present experimental program derive—they will not be further investigated in this work, as they are established data [7]. In fact, these are mixtures already being used to print full-size prototypes. With this experimentation, the WASP intends to take a step forward with respect to its first line of research and the current scientific literature on 3D printing, which is still focused on the technological aspects of the printability of earthen mixtures [3,8,9]. To the best of the authors’ knowledge, this is actually the first scientific work dealing with the long-term mechanical properties of biostabilized earthen mixtures for 3D printing.

The purpose of the experimental campaign presented in the following sections is to verify whether the use of finer portions of organic material improves the mechanical properties of earthen mixtures, as well as their workability. To this end, we performed uniaxial compression tests on cubic specimens made with shredded rice husk—the vegetable fiber of the mixture—and compared the results with those of cubic specimens made with nonshredded rice husk, with all components and dosages being equal (Section 3.1). This work is actually the first part of a wider experimental campaign, which includes the mechanical characterization of a 3D-printed wall segment, made with one of the mixtures of this first part [7]. Since the two parts of the experimental campaign are complimentary, where necessary, we will discuss the results of this first part with reference to the findings of [7]. Since the experimental campaign is the result of a long period of study of the traditional earthen construction techniques, on the one hand, and of the physical–mechanical properties of earthen mixtures for manual construction and 3D printing, on the other hand, this introductive section includes some subsections in order to provide a comprehensive overview of the knowledge and motivations behind the experimental campaign.

The compression tests took place at 90, 180, and 450 days of curing (Section 3.1) in order to verify how the mechanical properties vary over time. The relatively long observation period—rather rare in the scientific literature, as far as the study of earthen materials is concerned—allowed us to highlight some unexpected trends over time in the stiffness and behavior under cyclic loads. In particular, the effect of carbonation on earthen mixtures seems to have characteristics similar to those of the vulcanization of natural rubber (Section 4.2). This unexpected result, not highlighted in any previous experimental campaigns, can have several interesting repercussions for the technical practice of the 3D printing of earthen materials for construction purposes, if confirmed over longer periods of observation.

### 1.1. The Use of Raw Earth in Traditional Construction Techniques

Earthen construction (i.e., the construction of structural units manufactured from soil) is a vernacular solution that developed over many thousands of years on all of the inhabited continents of the world. The earth is indeed the oldest and most abundant of the construction resources. Being also cost-free, it had a particular development in rural regions and in regions where other traditional materials, such as timber and stone, are not available or affordable. This gave rise to many different and varied forms of construction techniques and applications, from the earliest known use of sun-dried compressed earth blocks (adobe blocks) in Jericho, Palestine (9000 BC), and onwards. The varied use of earthen construction resulted in earth playing a much wider, and arguably more significant, role as a building material than concrete over the course of human civilization.

While often synonymous with primitive or nomadic architecture, and incorrectly associated with poverty and vulnerability [10], earthen construction can also apply to certain types of architecture in developed countries and urban societies. Furthermore, the use of earthen constructions does not only concern the dawn of civilization, in the distant past. In fact, it has returned as a topical issue, starting from the early part of the twentieth century and driven by a growing interest in earthen materials in the field of pavement geotechnics [11]. The decisive push towards the modern development of earthen constructions came shortly after, and it originated in the need to preserve some historical sites that have been handed down to us, thanks to the ability of the compact earth to resist wear for centuries [12]. Additionally, materials professionals and researchers looking for lower-energy (and carbon dioxide) alternatives have increasingly returned to earthen construction since the 1970s. Indeed, earthen structures are sustainable forms of construction because of their characteristically low carbon footprint, which means they require lower carbon emissions for construction [13]. Moreover, earthen materials have less embodied energy than fired bricks or concrete, which means they need a fraction of the energy required for the manufacturing and processing of an equivalent amount of fired bricks or concrete. In the specific case of cement concrete, this fraction is of the order of 1% [14]. At the scale of the building, this means that the energy embedded in earthen buildings is between 30% [15] and 50% [16], less than traditional brick cavity walls and reinforced concrete structures. Earthen buildings are also energy efficient, thanks to the synergy between thermal conductivity and the hygroscopic properties of earthen materials [17]. This allows earthen buildings to consume less operational energy than conventional structures. Finally, since the earthen materials undergo only the slightest modification during construction, they need less energy to recycle them [18].

Because of the renewed interest in recent times, scientific articles about earthen construction now appear regularly in leading international journals [19]. For further information, Table S1 collects a selection of scientific articles that have investigated the topic of earthen construction, published in the years from 1948 to 2019 [10,11,20,21,22,23,24,25,26,27,28,29,30,31,32,33,34,35,36,37,38,39,40,41,42,43,44,45,46,47,48,49,50,51,52,53,54,55,56,57,58,59,60,61,62,63,64,65].

### 1.2. The Problem of Soil Stabilization in Earthen Construction

The raw material for earthen construction is subsoil, locally sourced and mixed with water and, occasionally, with other materials, such as straw or animal dung. The inclusion of straw or organic compounds stabilizes the earthen structures, improves their durability, and ensures their longevity.

Both the compressive and tensile strengths of the subsoil mixed with water (without the addition of other components) are relatively low, compared to many materials of the industrial age. To provide an order of magnitude, the uniaxial compressive strength of a soil mixture is generally less than 5 MPa and is often less than 1 MPa [66], while the uniaxial tensile strength is so low that it is often ignored in design. Therefore, in order to withstand the vertical and lateral loads imposed on them, the earthen structures must be massive; that is, they must have relatively thick walls. Since massive constructions are very expensive, earthen structures are not competitive in modern construction. To be precise, in order to compete with conventional fired bricks, the wet compressive strength of earthen blocks should be between 1.5 and 3.5 MPa, and the minimum compressive strength required for one-story external walls of a 300-mm thickness of rammed earth is 2 MPa [66]. This led to the need to increase the mechanical properties of earthen structures by means of the soil stabilization technique [21,35,36,45,46,51,52,53,54,55,67].

One of the oldest remedies to stabilize earthen structures is the use of natural fibers (straw). This is the case with cob, a traditional form of wall building consisting of successive layers of clay soil mixed with straw, stacked and molded in situ, generally without the need for formworks. The addition of vegetable fibers, such as straw, reduces the moisture movement and cracking of earthen constructions [68]. Recent experimental studies [69] have shown that embedded fiber lengths and moisture content are indeed important factors for improved strength. Additional remedies to improve the mechanical properties of the soil in historical earthen structures are mechanical tamping, and biomaterial additions in the form of egg whites or proteins [70,71,72].

The plural term, “biostabilizers”, is the cumulative name given to the biological products suitable for improving the properties of the soil, and “soil biostabilization” is the set of corresponding biological processes. Over the past century, industrial stabilizers, such as lime and Portland cement, have replaced the biostabilizers as additives to improve the strength and durability of earthen products, as well as to also control the moisture movements. However, adding cement affects sustainability [72], as it leads to an increase in the embodied energy, the carbon footprint, and the operational energy, and reduces the potential for recyclability [18,73,74]. For example, earthen materials stabilized with 9% cement have a carbon footprint equivalent to that of fired brick or weak concrete [75]. Furthermore, since cement-stabilized earthen materials require an energy-intensive (noneconomical) process to be fully recycled, they are usually downcycled—which, in turn, is energy-expensive—or they are dumped into a landfill for construction waste [18]. Cement stabilization also involves a drastic reduction in the hygroscopic behavior of the stabilized rammed earth, which controls the indoor comfort and has a direct impact on air-conditioning [30,65]. On the other hand, as far as embodied energy is concerned, the cumulative energy demand of compressed stabilized earthen blocks is nearly half that of traditional fired bricks [18].

To prevent chemical stabilizers from deteriorating the ecological credentials of earthen materials, some researchers propose the use of microbial-induced calcite precipitation (MICP) [76,77,78] and biopolymers [66,79,80,81,82,83,84,85,86,87,88,89] as potential alternatives to energy-intensive and CO_2_-producing stabilizers [90]. This has led to a renewed interest in biostabilization techniques. Both MICP and biopolymer stabilization are techniques of biocementation, which is one of the eight categories of soil biostabilization (Table S2 [91]), and which consists of binding the soil particles to increase the shear strength. Biocementation is, in fact, the most suitable category of biostabilization to replace chemical stabilizers in earthen construction materials.

Among the natural materials suitable for increasing the mechanical properties of earthen buildings, rice husk (RH) plays an important role because of its high silica content and low alumina content. The most common use of RH is in the form of rice husk ash (RHA), which has a silica content between 85% and 95%, and an alumina content between 0.5% and 2%. Adding some RHA increases the silica/alumina (Si/Al) ratio of geopolymeric binders, which leads to higher compressive strengths [92,93]. It is worth noting, however, that the Si/Al ratio has an upper bound, beyond which a further increase in the Si/Al ratio leads to a decrease in the compressive strength of the geopolymeric binders [94,95]. Indeed, an excess of silicate hinders the evaporation of water and the formation of silicon–oxygen–silicon bridge bonds (Si–O–Si bonds) during the geopolymerization process [96].

### 1.3. Additive Manufacturing of Earthen Buildings

One of the pioneering companies in the production of 3D printers for earthen structures is the Italian WASP, whose goal is to build transportable and energy-efficient 3D printers for zero-kilometer houses. The first success of the WASP in this sense came in 2015 with the construction of BigDelta (Figure S1), a 12-m high 3D printer for the on-site construction of earthen houses [97,98]. The BigDelta requires only one hour of three-person work to assemble, and a few meters of solar panels to power. In 2018, the WASP abandoned the idea of a giant 3D printer in favor of a modular collaborative 3D-printing system (Crane WASP, Figure S2, Video S1) that reinterprets classic building construction cranes from a digital manufacturing perspective. This allowed the WASP to create Gaia (Figure S3, Video S2), a 30-square-meter prototype made up of a 40-cm thick circular envelope (Figure S2). Gaia required 100 h of printing (in 2018). On the basis of Gaia’s experimental data, it is possible to imagine new economic scenarios, in which one hectare of cultivated paddy field is able to produce 100-square-meters of built area. Thanks to the honeycomb structure of the envelope, with rice husk that fills the cavities close to the inner shell of the envelope (Figures S2 and S4), Gaia does not need either heating or air conditioning, as it maintains a mild and comfortable indoor temperature in both winter and summer. The limit of the envelope of Gaia consists of it not being load bearing. In fact, eight wooden pillars support the flat roof (Figures S5 and S6), which is also made of wood and is equipped with lime and rice husk insulation. This limit has been overcome thanks to the collaboration between the WASP and Mario Cucinella Architects, which resulted in the double-dome structure of TECLA—Technology and Clay (Figures S7 and S8)—the first 3D-printed ecosustainable house model made entirely of local raw earth (completed in 2021 in Massa Lombarda, Italy) [99]. The double-dome solution made it possible to cover the roles of the structure, roofing, and external cladding at the same time, which also included the 3D printing of some interior furnishings (Figure S9). TECLA is the first housing module made using two Crane WASP collaborative printers at the same time (Video S3). The WASP also developed software capable of synchronizing the two extruder arms in order to avoid collisions and guarantee simultaneous operation.

With the Gaia and TECLA prototypes, the WASP undertook two different lines of research. The first line of research deals with the 3D printing of infill walls using natural mixtures of local origin, without any type of chemical stabilization (Gaia). The second line of research aims at the 3D printing of load-bearing elements by additivating the mixtures with the minimum quantity of hydraulic lime-based stabilizers, which allows for the creation of a new circular building model, made entirely with reusable and recyclable materials.

## 2. The Idea behind the Experimental Campaign

The experimental research on earthen mixtures for additive printing (Section 3) was born by combining the results on the use of silica in geopolymeric binders (Section 1.2), the biocementing capacity of biostabilizers (Section 1.2), and the construction technique of cob (Section 1). As in cob, the vegetable fiber of the experimentation (in our case, the rice husk) is in the natural state, which avoids the reduction to the state of ash by combustion. Thanks to its high silica content (Section 1.2), the rice husk (RH), used together with lime, forms a natural biocomposite that is capable of improving the mechanical properties of earthen mixtures through biocementation (Section 1.2). In particular, the lime suitable for use in combination with RH—as well as with any other vegetable fiber—is aerial lime, and not hydraulic lime. While the reaction of the hydraulic lime (which also hardens in water) slows down when it loses its water content, the reaction of the aerial lime (which hardens in air) begins just when it loses water and continues indefinitely (hardening progresses slowly). This allows the biocementation effect of the biocomposite to have an unlimited action over time, contributing more and more to the strength of the structure. The use of aerial lime also allows the biocomposite to manage air humidity very well.

The combination of a vegetable fiber with a mineral (in our case, aerial lime) and the water allows the vegetable fiber to mineralize and become resistant to bacteria, molds, insects, rodents, and fire, resulting in very durable materials. In the specific case of aerial lime, the mineralization of the fiber occurs during the carbonation process (Figure S10). The product of lime carbonation is calcium carbonate (CaCO_3_), a salt that is also the main component of the rock used to produce aerial lime (limestone). This closes the so-called “lime cycle” (Figure S10), which starts from calcium carbonate—transformed into calcium oxide (quicklime), then into calcium hydroxide (hydrated lime, or slaked lime)—and ends in contact with the carbon dioxide of the air (carbonation), again in calcium carbonate.

The production of calcium carbonate that follows from the carbonation of lime over time is an effect somewhat similar to the delayed precipitation of calcium carbonate that takes place in MICP (Section 1.2), which occurs because of the metabolic action of bacteria (also unlimited in time). The presence of calcium carbonate at the end of the lime cycle is the reason for the increased mechanical properties of earthen buildings made with a little lime in the mixture. In fact, the calcium carbonate crystals produced by carbonation have good power of adhesion, both to each other and to the other elements in the mixture, which results in the cementing of the hardened product.

Thanks to the cyclic structure of the lime transformation process, the RH–lime biocomposite respects the environment. If it were necessary to demolish an earthen building made with RH–lime biocomposite, the calcium carbonate would be completely recyclable and reusable in new earthen mixtures. In the event that calcium carbonate is not reused, it can remain on the ground without causing soil contamination, as it does not contain industrial chemicals.

Similar to the method used by [100] to increase the pozzolanic activity in geopolymers, the idea behind the experimental campaign discussed in the following sections is to make the most of the cementing capacity of the biocomposite by shredding the RH. In fact, all other conditions being equal (including the quantity of RH), shredding will increase the contact surface between the RH and the earthen matrix, favoring the formation of Si–O–Si bonds.

The use of RH in earthen mixtures for additive printing paves the way for new technological applications that can help reduce one of the most serious environmental hazards. In fact, every year, rice cultivation produces a large volume of RH (from 20% to 25% by weight of the rice crop), which is used only to a small extent [101], despite being a good source of renewable energy [102]. Most of the RH is treated as organic waste, and it undergoes disposal in landfills or through open combustion [103]. Both actions are harmful to the environment: landfilling may potentially pollute the soil and aquifers, and burning outdoors increases the amount of carbon dioxide in the atmosphere, thereby contributing to global warming.

## 3. Materials and Methods

### 3.1. The Two Earthen Mixtures of the Experimental Program

**Figure 1:**
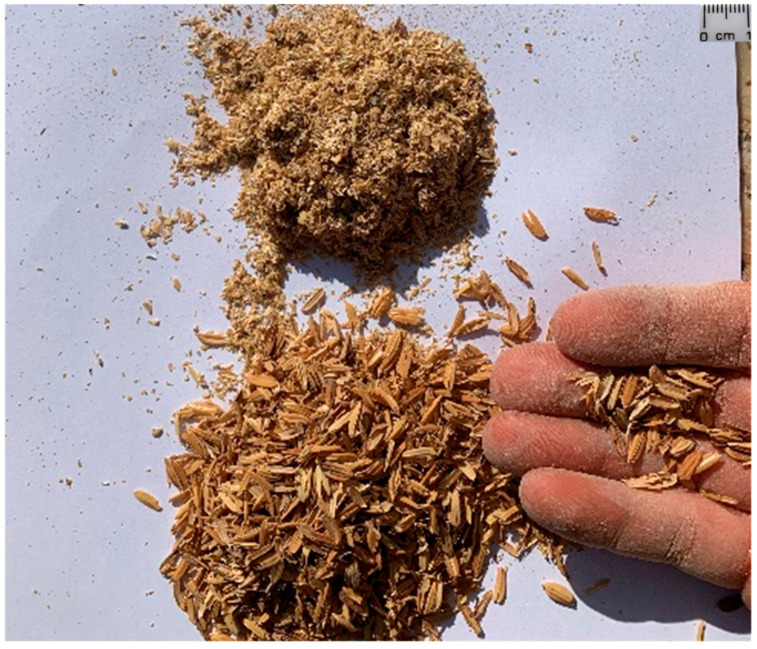
The two types of RH used in the experimental program, arranged by decreasing lengths from bottom to top: unaltered RH (maximum size variable from 6 to 8 mm), and shredded RH (maximum size: 2 mm).

In the spirit of the idea behind this experimental program (Section 2), the two mixtures under study differ only in the length of the vegetable fiber, which is the RH. In particular, the mixture referred to as the “TQ mix” contains unaltered RH, while the mixture referred to as the “LT mix” contains shredded RH (Figure 1).

The mix design of the experimental program is the result of many years of the WASP research on the earthen mixtures suitable for the 3D printing of buildings (Section 1.3 [104]). The preparation of the mixtures and specimens took place at the WASP headquarters (Massa Lombarda, Italy). The TQ and LT mixes have the compositions by weight shown in Table 1.

- The soil is of local origin, as it is the soil excavated on site at the WASP headquarters (Massa Lombarda, Italy), at a depth of 50–150 cm. The selection of the soil to remove stones and other foreign bodies took place at the time of excavation, using a screening bucket (grain size: 0–6 mm, screening cylinder modified by the WASP, with additional holes and flanges to optimize the rolling and filtering dynamics). The soil classification then followed the steps shown in Figure S11. From the analysis of a soil sample, it emerged that the composition of the soil consists of 30% clay, 40% silt, and 30% sand. It is, therefore, a silty clay soil, which is the optimal soil to meet the workability criterion of a mixture for 3D printing (as for cob). Furthermore, the organic content of the soil (ASTM D2974-07a: 2012) is 3.7%. According to the Highway Research Board (HRB)/UNI EN ISO 14688-1:2018 soil classification, this soil is of Class A-4. Its optimum dry density is 1815 kg/m^3^ (EN 13286-2: 2005). Table 2 shows the results of the grain size analysis performed on the soil sample.
- The lime-based binder is a high-performance fiber-reinforced powder stabilizer with hydraulic action for the treatment and consolidation of soils and recycled, or first-use, aggregates. Its composition includes hydraulic lime for 25–50%, and hydrated lime (Section 2) for 20–25%. The presence of specially selected mineral additions with pozzolanic activity (not deriving from the use of cement) and having binding properties (>22% by weight) significantly increases the durability of the hardened mixture, as well as the resistance to the leaching of the stabilized material. In addition, the polypropylene fibers present in the product (dosage ≥ 0.1%), although with an aspect ratio greater than 600, are easily dispersed in the mixture and improve the final mechanical performance of the treated soil.
- Both the hydraulic lime contained in the lime-based binder and the one added separately have the function of allowing the carbonation to begin when the mixtures are still in their fresh state, which reduces the setting times. In fact, since the aerial lime hardens in contact with the CO_2_ contained in the air (Section 2), in the absence of hydraulic lime, the carbonation would begin only after the drying of the mixtures. Having a carbonation that begins immediately after mixing with water is, instead, mandatory in order to anticipate the setting of the material to meet the buildability criterion—the second requirement in 3D printing (Section 1)—more quickly. It is worth remembering that, in fact, each printed layer must be strong enough to withstand the weight of subsequent layers before hardening and before achieving some degree of structural integrity [9]. The faster the material sets, the faster the printing process can proceed, which reduces production times and costs. When the mixtures dry, the hydraulic lime exhausts its function, and the carbonation continues thanks to the fraction of aerial lime.
- The (wet) silica sand added to that already contained in the soil has a fluvial origin. Its grain size is between 0.00 and 0.60 mm.

The kneading of the two mixtures took place by means of the muller, which is capable of making the compounds homogeneous and workable.

As mentioned in Section 1, we did not carry out specific tests to verify the extrudability, buildability, and workability of the two mixes because these are topics of previous scientific works, belonging to the first line of research of the WASP (Section 1.3), which the WASP team either wrote with their own hands [104] or published in collaborations with other authors [105]. Extrudability, buildability and workability are, in fact, technological aspects related to the 3D-printing process, and, for a 3D-printer manufacturer such as the WASP (Massa Lombarda, Italy), they come first. Therefore, the WASP has progressively refined them over time, starting with its first 3-D printing experiments in 2017. The mix designs in Table 1 are precisely the result of the WASP’s long work on perfecting the technological aspects of the 3D-printing process. To be precise, the development of both the TQ mix and the LT mix took place during TECLA’s printing research. The success of these preliminary tests paved the way for the definitive printing of TECLA, the WASP’s second full-scale housing prototype (Figure S8), and, for this reason, they are proof of the feasibility of the entire process, from the architectural design and soil supply to the 3D printing of entire housing modules (Figure S11).

In this paper, we will present the results on the trend over time of the mechanical properties of the two hardened mixtures subjected to uniaxial compressive loads. In fact, since the characterization of the mechanical properties of earthen mixtures for 3D printing belongs to the second line of research of the WASP (Section 1.3), born later, this aspect is still under study, particularly with regard to the behavior over time.

**Figure 2:**
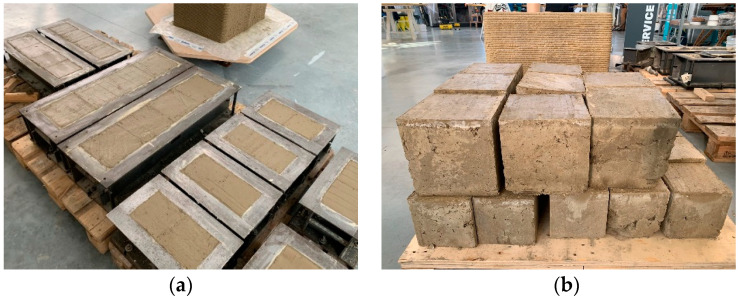
Preparation of the specimens: (**a**) the specimens after casting in the formworks; (**b**) appearance of the specimens upon removal of the formworks (before the selection that led to the exclusion of some specimens).

The specimens subjected to the load test (Table 3) have a cubic shape (Figure 2), with a side of 150 mm. Since printability is not the subject of this work, the production of the specimens did not make use of mechanical extrusion, but of hand compaction inside the cubic formworks (Figure 2a). This does not affect the validity of the results obtained. In fact, the extensibility of the results obtained for specimens with hand compaction to the behavior of the 3D-printed solids derives from the comparison between the results of this paper (Section 4) and its complementary experimentation on a 3D-printed wall segment made with the LT mix [7]. The earthen wall segment of [7] is partially visible behind the cubic specimens in Figure 2: in the fresh state (the day of 3D printing) in Figure 2a, and in the hardened state in Figure 2b. To allow for the comparison between the experimental results of the two complementary studies, the 3D printing of the wall segment took place on the same day, and with the same mixture, as the casting of the LT cubic specimens. Moreover, the uniaxial compression test on the 3D-printed wall segment took place on the same day as the uniaxial compression tests on the LT1, LT2, and LT3 specimens, providing similar values of compressive strength (Section 4.1 [7]). This means that the extrusion process did not substantially change the strength of the earthen material along the direction orthogonal to the layers, compared to that of the hand-compacted material. For everything concerning the anisotropic behavior of the 3D-printed solid made with the LT mix, and the bond between the extruded layers under the compression load, we refer the reader to what we have already written in [7].

After the removal of the formworks (Figure 2b), curing took place under controlled conditions of temperature and humidity in the curing chamber. In particular, as there are still no specific regulations on the preparation of specimens made with earthen mixtures, we followed the indications of temperature and humidity provided by UNI EN 12390-2: 2009 for the curing of the mortar and concrete specimens: a controlled temperature of 20 ± 0.5 °C, and a relative humidity ≥95%.

**Figure 3:**
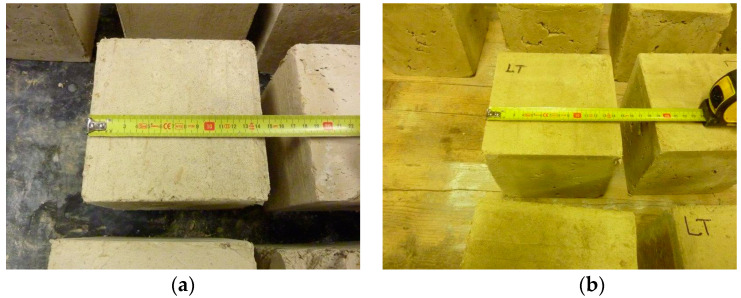
Dimensional checks after drying: (**a**) TQ specimens; and (**b**) LT specimens.

Both the TQ and the LT specimens showed shrinkages of approximately 5 mm per edge (Figure 3) when completely dry. To be precise, the average (negative) percentage variation in the linear dimensions due to shrinkage was 2.44%.

The average density after shrinkage was 1446.360 ± 38.806 kg/m^3^ for the TQ mix, and 1446.687 ± 35.686 kg/m^3^ for the LT mix, with the ξ—the maximum percentage variance (in absolute value)—equal to 3.4% for the TQ mix, and to 3.3% for the LT mix (Table 4):

(1) $$\xi =\frac{max\left|\rho_{max}-\bar{\rho };\rho_{min}-\bar{\rho }\right|}{\bar{\rho }},$$

where:

- $\rho_{max}$ is the maximum density of the specimens made with the same mixture;
- $\rho_{min}$ is the minimum density of the specimens made with the same mixture;
- $\bar{\rho }$ is the average density of the specimens made with the same mixture.

The small value of the ξ in the dry state indicates that the manual compaction was carried out in a sufficiently accurate way not to cause inhomogeneity between the specimens made with the same mixture.

We subjected the eighteen specimens of the experimental program—nine for each mixture (Table 3)—to uniaxial compression tests in groups of three, at 90, 180, and 450 days of curing (the casting of all specimens took place on the same day). Section 4 will show the results of the uniaxial compression tests, averaged over the three different curing periods.

### 3.2. Test Setup

As with concrete and masonry units, the most commonly used parameter to evaluate the performance of an earthen construction material is the compressive strength [25,45,106,107], which is the maximum stress value a material can withstand when subjected to uniaxial compression. The routine tests to determine the compressive strength of an earthen construction material are direct unit strength tests, RILEM (1994) tests, and indirect tests [25]. It is worth noting, however, that the choice of the testing procedure affects the test results, making it impossible to obtain a unique compressive strength value. Therefore, similar to what happens with concrete and masonry specimens [108,109,110,111], the compressive strength of an earthen construction material requires an adequate identification procedure [25]. Furthermore, the compressive strength of an earthen construction material depends on the geometry of the specimen (cylinder, prism, or cube), as well as on the dimensions and the aspect ratios [25,106,107]. In short, the earthen construction material suffers from the shape effect, the well-known behavior of brittle materials (in general) and of concrete (in particular) [112]. The shape effect also affects the branch beyond the load peak (softening branch), under both static [109,113] and dynamic [114] load conditions. The correct evaluation of the shape effect is also a major concern in modeling earthen construction material, as with all brittle (heterogeneous) materials [115,116]. In the specific case of the TQ and LT specimens, the shape effect did not generate any problems in the comparison of the experimental data, since the fresh samples were all of the same shape, dimension, and aspect ratio. However, a further geometric effect had a decisive influence on the results of these experimental tests, namely, the geometric imperfection induced by shrinkage during the drying phase (Figure 3).

**Figure 4:**
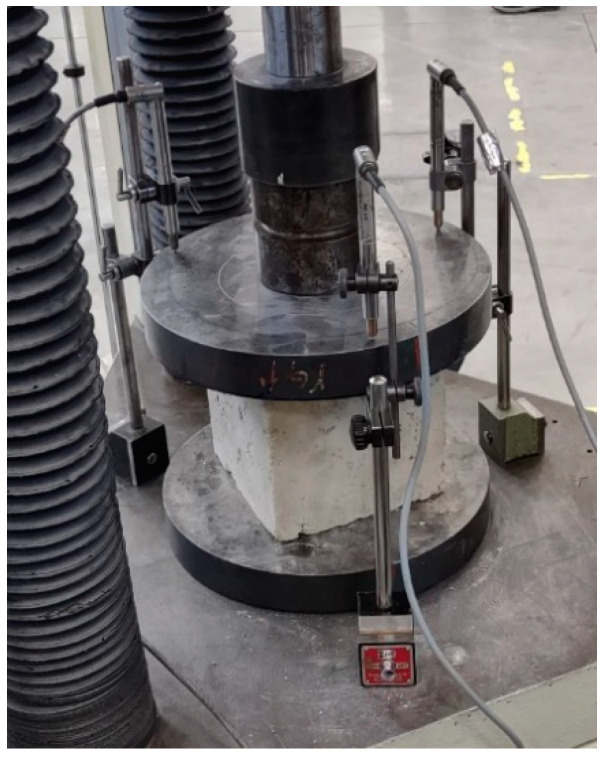
Placement of the three LVDTs on the bottom plate of the testing machine.

The test method chosen to determine the compressive strength of the TQ and LT specimens is the uniaxial compression test (Figure 4), conducted in the displacement control mode at the LiSG laboratory of the University of Bologna. The displacement speeds were 0.01 mm/s apart in the initial and final stages of the stabilization cycles (Section 3.3), which required reversing the direction of the motion of the testing machine head.

In order to reduce the effect of the geometric imperfections on the experimental data, we acquired the vertical displacements of the upper faces of the specimens with three LVDTs (linear variable differential transformers) produced by Gefran SPA (Brescia, Italy), positioned radially at 120° on the bottom plate of the testing machine (Figure 4). The vertical displacement is then the average value acquired by the three LVDTs. 

**Figure 5:**
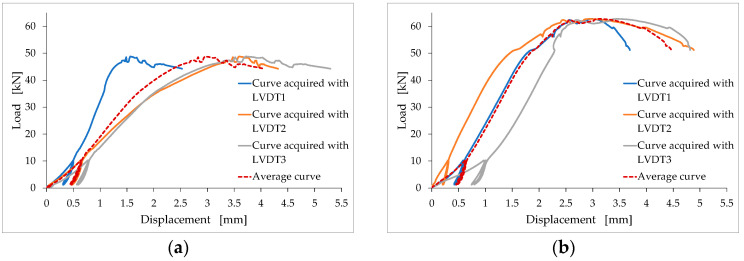
Significant rotations of the upper face under uniaxial compression: (**a**) TQ12 specimen; and (**b**) LT9 specimen.

The choice of placing three LVDTs in different positions is justified by the effect that geometric imperfections have on the acquisition of the displacements. It is worth noting, in fact, that shrinkage does not occur equally along all directions. As a result, the top and bottom faces of the specimens are no longer parallel after the mixture has hardened. This implies that the vertical load is not in the axial direction, and that it induces a bending component that causes the faces of the specimens to rotate during the compression test (to different extents, from specimen to specimen). The rotation of the upper face is actually an inevitable effect that would also occur in the case of perfect parallelism. In fact, it occurs even in rectified concrete and masonry specimens. In the case of using one LVDT, the combined effect of the rotation of the faces and the distance of the LVDT from the axial position thus alters the acquisition of the displacements, providing incorrect values (Figure 5).

Conversely, the average of the values of three equidistant LVDTs provides the displacement for the points of the axis of the specimen (axial displacement), and is, therefore, not affected by rotation. Figure 5 shows the effect of rotation on the displacements for the TQ12 and LT9 specimens, which are the specimens for which the rotation of the top face was most evident. The comparison between the curves highlights the extent of the error committed if, instead of using three LVDTs, only the LVDT in Position 1 had been used for the TQ12 specimen, and only the LVDT2 or LVDT3 for the LT9 specimen.

### 3.3. Identification of the Stiffness

The second parameter that characterizes earthen construction materials is stiffness, namely, the extent to which an object resists deformation in response to an applied force. In brittle materials, there are actually several ways to estimate stiffness from experimental data. Indeed, it can be either the slope of the stress/strain curve at any specified stress or strain (tangent stiffness, or tangent modulus), or the slope of the straight line connecting the origin of the stress/strain curve with another point on the curve (secant stiffness, or secant modulus). Among all the tangent moduli, moreover, the tangent modulus at the origin (initial stiffness) is of particular significance. This value is, in fact, associated with the behavior of the material for low load values, a behavior that is linear elastic for most of the brittle materials. Therefore, the tangent modulus at the origin is equivalent to Young’s modulus in the elastic (reversible) deformation regime. Usually, the secant modulus is, instead, a percentage of the Young’s modulus and describes the stiffness of a material in the inelastic region of the stress/strain diagram.

To evaluate the tangent modulus at the origin correctly, it is of fundamental importance to have reliable experimental data for low load values. However, one of the main reasons for uncertainty in the experimental data derives from the coupling effect that characterizes the first phases of the compression test on brittle materials [113]. This phenomenon results from the adaptation of the plate of the testing machine to the upper face of the specimen, which typically suffers from geometric imperfections. The adaptation results in a rotation of the plate of the testing machine, which occurs only during the coupling phase. It is, therefore, a different phenomenon from that described in Section 3.2, even if both give rise to rotations.

**Figure 6:**
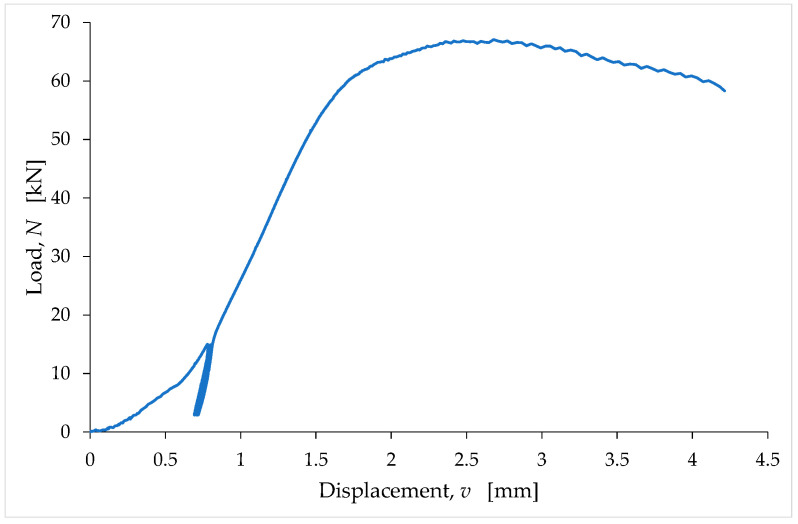
Acquired N/v curve for the LT6 specimen (180 curing days).

The rotation of the plate of the testing machine during the coupling phase affects the experimental result to such an extent that it cannot provide a constitutive meaning to the initial part of the load (N)/displacement (v) curve [113]. To be precise, the coupling phenomena distort the N/v curve, altering its shape in earthen materials (Figure 6), as in all brittle materials.

Since these phenomena occur at the beginning of the load test, when the material is still in the linear elastic field, the alteration of the experimental data concerns the part of the N/v curve near the origin, and results in the loss of the direct proportionality between the load and the displacement. Consequently, the first branch of the acquired N/v curve does not have a linear trend. Instead of the linear branch, the acquired N/v curve shows a branch with an increasing slope, that is, with upwards concavity (Figure 6). In fact, during the coupling phase of a common brittle material, the slope of the N/v curve increases up to an upper bound value, which remains almost constant for a short interval of the displacement values. After this short interval, the slope decreases continuously until the end of the uniaxial compression test.

It is common practice to reconstruct the initial part of the N/v curve with a conventional straight-line segment, whose slope is equal to the slope reached by the N/v curve when the coupling effects are exhausted. Therefore, the average value of the slope in the interval in which it is almost constant (before it begins to decrease) is the suitable value to reconstruct the initial part of the N/v curve.

The reconstruction procedure of the experimental data is a very delicate operation, as it depends on several parameters. For example, aspects such as the speed of the execution of the test, the quality of the acquisition tools, the speed of data acquisition, as well as the imperfections of the specimen are decisive. The combination of all the experimental uncertainties results in an acquired N/v curve that is not smooth, but that shows an oscillatory behavior that depends on the individual test. This poses the problem of how to identify the slope function of the acquired N/v curve, that is, its first derivative, $N^{\prime }\left(v\right)$.

Proceeding analytically is not easy, as the shape of the N/v curve makes it difficult to find a function that approximates it along its entire length. To simplify the procedure, it is necessary to select only a part of the data, extracted from the initial section of the curve. However, this requires the direct intervention of the investigator and does not allow the identification process to be fully automatic.

**Figure 7:**
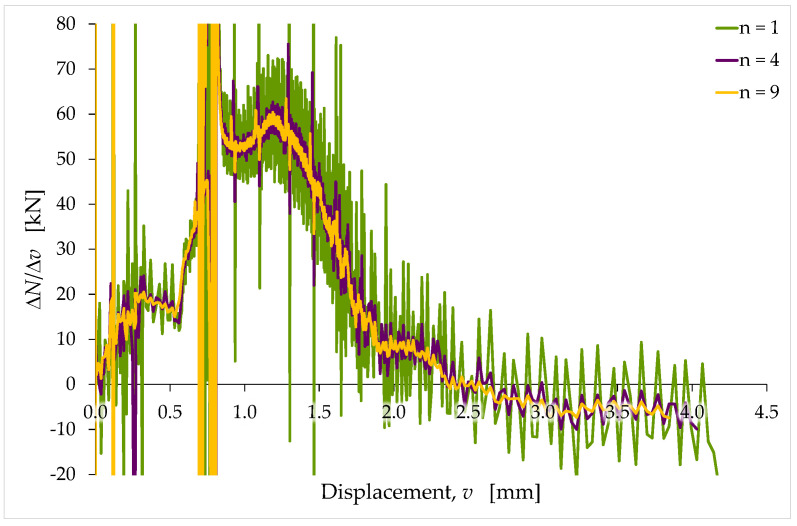
Identification of the first derivative of the *N*/*v* curve as the ΔN/Δv ratio (LT6 specimen): the vertical lines in the range 0.7≤v≤0.8 derive from the infinite value reached by the slope along the unloading–reloading cycles of Figure 6.

Since the intervention of the investigator is unavoidable in any case, it is easier to proceed manually, identifying the slope function, $N^{\prime }\left(v\right)$, as the ΔN/Δv ratio (Figure 7):

(2) $${N^{\prime }|}_{v=v_{i}}=\frac{\Delta N\left(v_{i},n\right)}{\Delta v\left(v_{i},n\right)}=\frac{N_{i+n}-N_{i}}{v_{i+n}-v_{i}},$$

where:

- i is the index of the displacement value at which to calculate $N^{\prime }$. It also sets the first end of both the displacement range and the load range for the slope calculation;
- n is a natural number >0. It sets the position of the second end—in both the displacement range and the load range—since the two ends of the displacement and load ranges for the slope calculation are not necessarily consecutive data.

Because of the small quantities involved in Equation (2), and the oscillatory nature of the acquired N/v curve, the manual identification procedure results in oscillatory slope values. It is possible to reduce the fluctuations of $N^{\prime }\left(v\right)$ by increasing the value of n (Figure 7). In any case, the optimal value of n depends on the individual test. Therefore, it requires an accurate evaluation by the investigator.

In order to favor the coupling between the specimen and the testing machine, it is possible to carry out some stabilization cycles [113], which consist of repeated unloading–reloading cycles performed for a fraction of the presumed maximum load (Figure 6). The effect of the stabilization cycles is to shorten the coupling phase, which means that the adaptation phenomena cease for a lower value of the applied load. If the stabilization process is successful, the slope of the load/displacement curve at the end of the last stabilization cycle is equal to the slope that the load/displacement curve would have shown in the absence of stabilization, at the end of the coupling phenomena. It is worth noting that the minimum load value in an unloading–reloading cycle is always greater than zero, as the total removal of the load would cause a loss of contact between the testing machine and the specimen. The choice of the load value at which to start the unloading–reloading cycles is also up to the operator and strongly depends on his experience. This value varies between approximately 10% and 20% of the presumed maximum load.

The unloading–reloading cycles are hysteretic cycles, with an accumulation of displacement at the end of each cycle, which is due to creep and other inelastic phenomena [111]. The execution of the unloading–reloading cycles ends when the incremental displacement accumulated at the end of a cycle is sufficiently small; that is, when it is lower than a predetermined maximum threshold. The number of unloading–reloading cycles in Figure 6 is equal to four.

**Figure 8:**
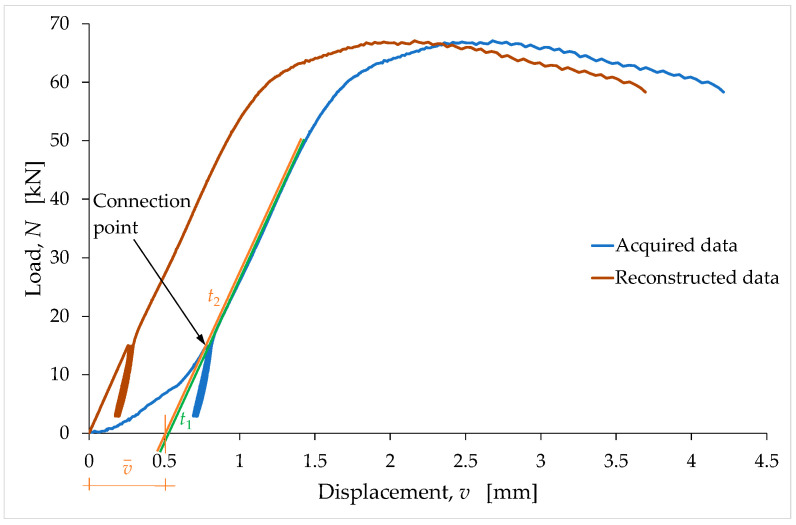
Identification of the slope for the replacement of the first branch of the N/v curve with a straight-line segment, and of the translation, $\bar{v}$, of the reconstructed and nonreconstructed branches: LT6 specimen.

As an example of the reconstruction procedure, Figure 8 compares the reconstructed curve to the acquired data for the LT6 specimen.

The straight line $t_{1}$, in Figure 8 is the tangent line at the end of the last unloading–reloading cycle. The slope of $t_{1}$ provides the slope of the reconstructed segment. In order to not lose the information on the slopes of the unloading–reloading cycles (Section 4.2), the connection point between the linear segment and the rest of the curve is the last point of the curve before the first unloading–reloading cycle (Figure 8). Therefore, the reconstructed straight-line segment lies on the straight line $t_{2}$, which is parallel to $t_{1}$, and which passes through the last point of the curve before the first unloading–reloading cycle (Figure 8).

Since the reconstructed curve must pass through the origin, the reconstruction procedure also involves the translation of the reconstructed and nonreconstructed branches along the negative semiaxis of the displacements. In other words, the reconstructed displacements are equal to the displacements of the reconstructed and nonreconstructed branches minus $\bar{v}$, which is the distance from the origin of the point of intersection between $t_{2}$ and the horizontal axis (Figure 8).

**Figure 9:**
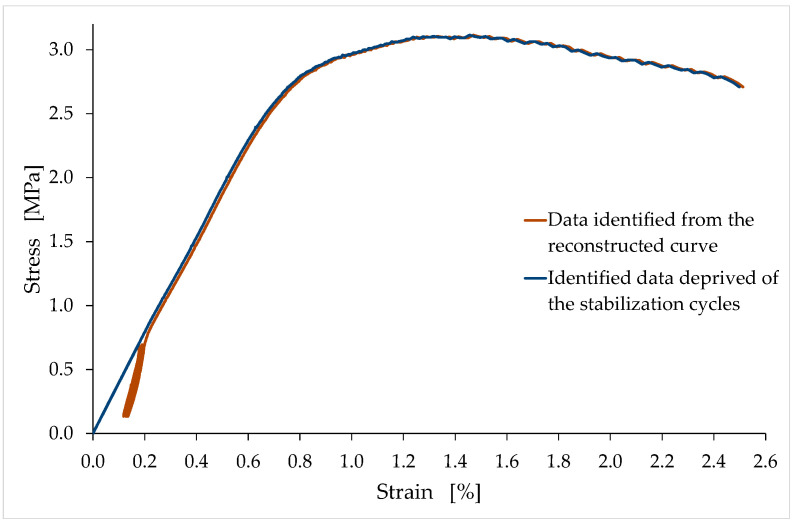
Elimination of the stabilization cycles from the stress/strain curve: LT6 specimen.

After the reconstruction of the first linear branch, the procedure for identifying the stress/strain curves proceeds with the elimination of the stabilization cycles (and, consequently, of the accumulated strain) to provide monotonic nondecreasing curves up to the maximum stress (see Figure 9 for the LT6 specimen). The connection point in this second phase of the reconstruction procedure is the final point of the last unloading–reloading cycle. The slope of the reconstructed branch in the stress/strain curve provides the tangent modulus at the origin.

## 4. Experimental Results and Discussion

### 4.1. Effect of RH Shredding on Compressive Strength

**Figure 10:**
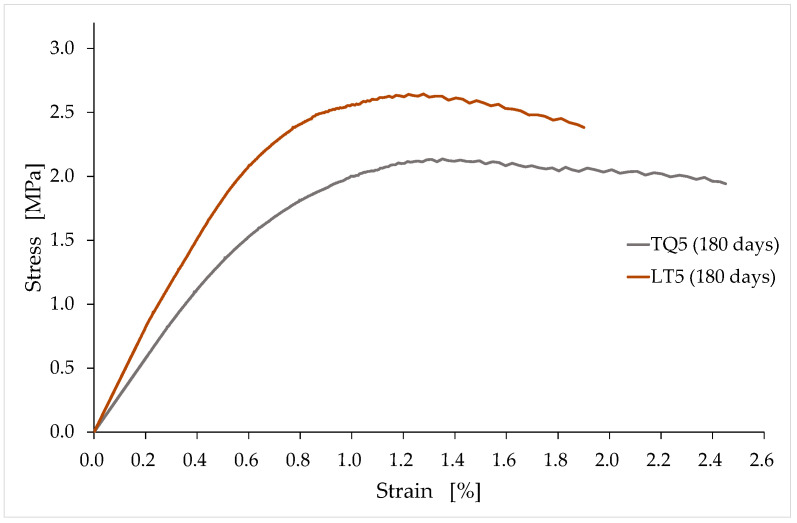
Comparison between the stress/strain curves of the TQ5 specimen and the LT5 specimen.

**Figure 11:**
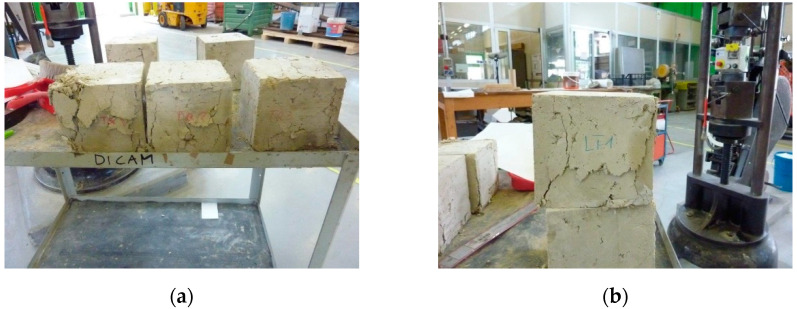
Appearances of the specimens after removal from the testing machine: (**a**) three TQ specimens in the foreground; and (**b**) detail of one LT specimen.

The behavior of the specimens under uniaxial compression load was of the brittle type, with pronounced softening branches after the maximum load (Figure 10). In particular—as is common for fragile materials—during the softening phases, the damage phenomena produced extensive crack patterns that were visible to the naked eye on the faces of the specimens. However, the specimens retained some degree of compactness, even when removed from the testing machine (Figure 11).

As anticipated in Section 1, the results of the experimental campaign allowed us to highlight some unexpected results, as well as to confirm the expected results. Among the confirmed expected results is that the softening branches are longer for TQ specimens than for LT specimens (Figure 10). In fact, the greater lengths of the vegetable fibers in the TQ specimens provide an increased binding effect, which allows for a better exploitation of the hardened mixture beyond the stress peak.

**Figure 12:**
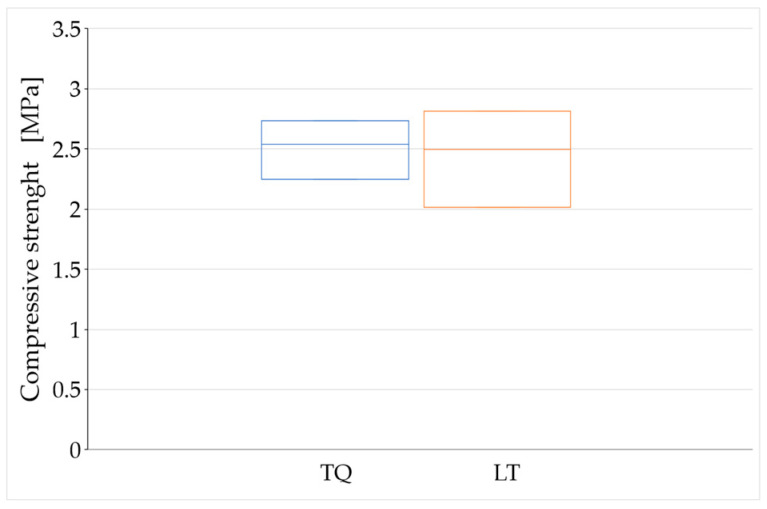
Minimum, average, and maximum values of compressive strength after 90 days of curing.

**Figure 13:**
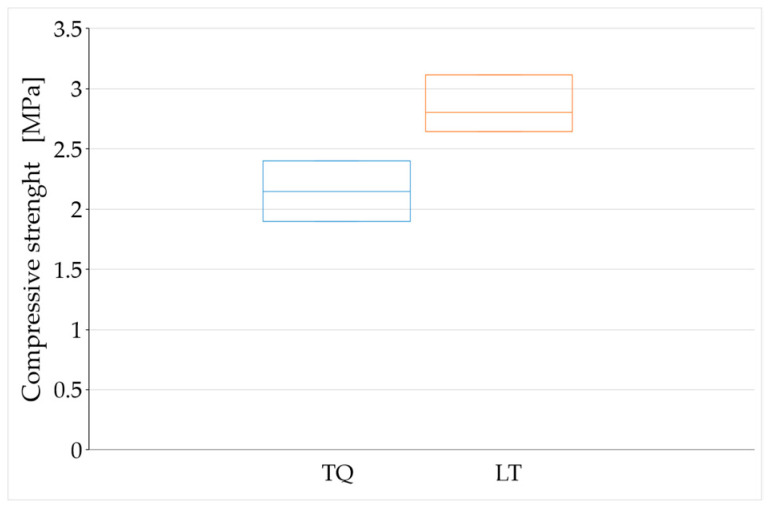
Minimum, average, and maximum values of compressive strength after 180 days of curing.

**Figure 14:**
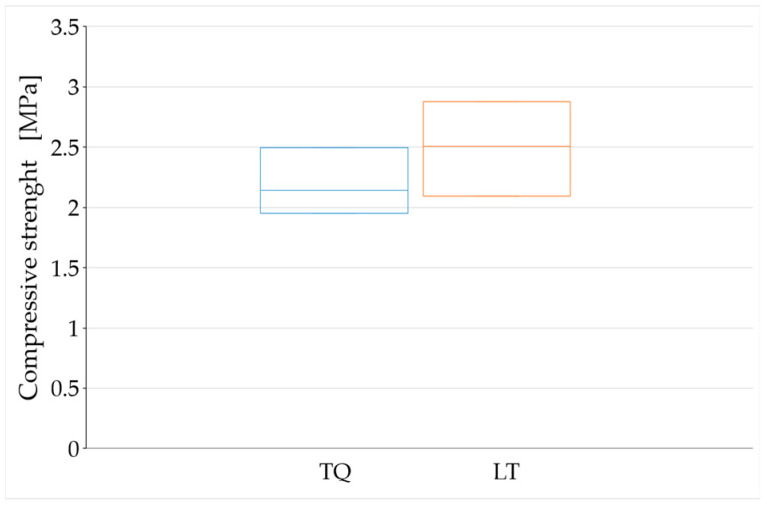
Minimum, average, and maximum values of compressive strength after 450 days of curing.

The average values of compressive strength after 90, 180, and 450 days of curing (Figure 12, Figure 13 and Figure 14) are higher than the minimum compressive strength required for one-story external walls of rammed earth (2 MPa, Section 1.2). As far as the effect of the RH length on the compressive strength is concerned, however, it is not possible to draw definitive conclusions because of the high dispersion of the data shown by these tests (Figure 12, Figure 13 and Figure 14).

The high variability of the compressive strength values is probably a consequence of the difficulties encountered in compacting the fresh mixtures inside the formworks, particularly for the LT specimens. This resulted in the nonoptimal filling of the formworks, with some air bubbles persisting on the contact surfaces between the two fresh mixtures and the formworks. Compaction was particularly problematic at the vertexes of some specimens (Figure 2b), which resulted in their exclusion from the experimental program. The final effect after the hardening of the remaining specimens is, in any case, that of a defectiveness that negatively influences the determination of the compressive strength.

The reason for the greater compaction difficulties in LT specimens compared to TQ specimens is that the shredding of the RH resulted in the need to wet a larger surface of RH. Since the volume of mixing water was identical for the two mixtures, this subtracted water from the mixture of the LT specimens, reducing their workability. The greater defectiveness of the LT specimens compared to the TQ specimens is particularly evident in Figure 12 and Figure 14, where the range of variability of the compressive strength is greater for the LT specimens than for the TQ specimens.

**Figure 15:**
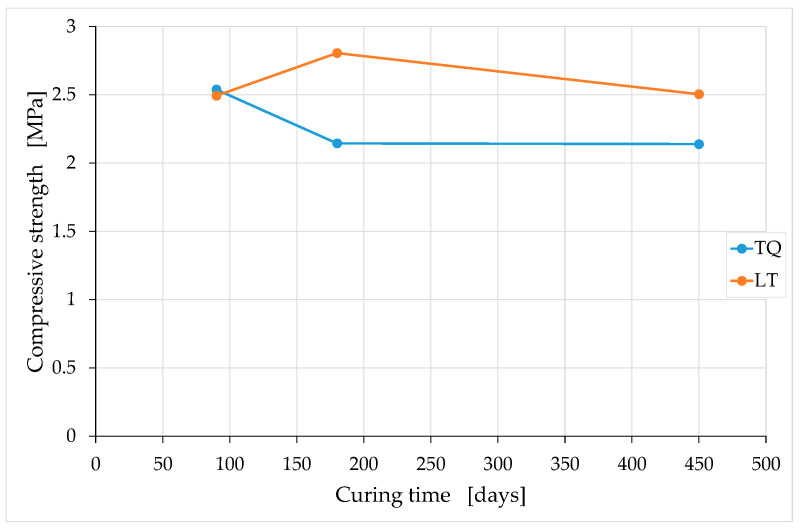
Trend over time of the average compressive strength values for the TQ and LT mixes.

For the aforementioned reasons, the average values obtained for the groups of specimens with the same curing time only provide indications about a trend that would deserve further investigation in a greater number of specimens. The variation of these average values over time (Figure 15) seems to show the weak sensitivity of the compressive strength to the RH particle size, particularly after long curing times. To be precise, the compressive strength after long curing times appears to be higher for the LT specimens than for the TQ specimens.

The higher compressive strength for the LT specimens compared to that for the TQ specimens is actually an expected result, as it is reasonable to assume that the greater contact surface (between the RH and lime) generated by shredding favors the formation of Si–O–Si bonds (Section 2), with an increased biocementing effect (Section 1.2). However, one should take this result with caution, as the difference in compressive strength is small and could fall within the uncertainty range due to the dispersion of the results.

A possible explanation of the modest increase in the compressive strength may be the fact that the contact between the RH and the lime does not occur directly since the lime is just one of the components of the earthen mixture. In other words, the contact takes place in different percentages between the RH and the various components of the earthen mixture, including a part of the lime contained in the mixture. Since not all the lime in the mixture comes into direct contact with the RH, a given increase in the RH surface does not produce an increase in the number of Si–O–Si bonds to the same extent, but, rather, by a smaller percentage. This means that, for the same quantities used, the number of biocementing bonds could increase by premixing the lime together with the RH immediately before inserting them into the mixture.

Another result that seems to emerge from Figure 15, albeit within the limits already stated, is that the compressive strength remains almost constant after a sufficiently long curing period. A similar behavior occurs in soft clays mixed with cement as a chemical stabilizer [117]. In particular, the authors of [118] found that the increase in the compressive strength of a clay–cement mixture is rapid in the first curing period, and that it slows down significantly over time. A few years later, the authors of [119] proposed a generalized equation to estimate the increase in the unconfined compressive strength with the curing time for clay–cement mixtures:

(3) $$\frac{q_{D}}{q_{28}}=0.039+0.283_{\;}ln\left(D\right),$$

where D is the curing time in days; $q_{D}$ is the strength at time D; and $q_{28}$ is the strength at 28 days. In the absence of previous specific studies on soil stabilization using the RH–lime biocomposite, Equation (3) is the most useful formula to provide a qualitative evaluation of the trend over time of the TQ and LT strengths. In fact, the local soil used for the preparation of the mixtures is a silty clay soil (Section 3.1), which is, precisely, a soft and creamy clay. Furthermore, because of the greater pozzolanic activity of cement compared to aerial lime, we can consider the increases in strength over time obtained using cement (as a stabilizer) as the upper bound of that obtainable with aerial lime.

With this in mind, we can use Equation (3) to estimate $q_{180}$ and $q_{450}$, that is, the upper bounds of the compressive strengths at 180 and 450 days:

(4) $$q_{180}=1.509q_{28},$$

(5) $$q_{450}=1.768q_{28},$$

from which it follows that the percentage increase in the compressive strength from 180 to 450 days of curing is equal to 17%:

(6) $$\frac{q_{450}-q_{180}}{q_{180}}=0.172.$$

Even the value provided by Equation (6) is an upper bound, as it is a consequence of both the main chemical reactions that regulate the behavior of the clay–cement mixtures, the primary hydration reaction between cement and water, and the secondary pozzolanic reaction between the lime released from the cement and the clay minerals [120]. Since the TQ and LT mixes benefit from only one of these chemical reactions—the reaction between lime and clay minerals—the expected percentage variation between 180 and 450 days of curing for the TQ and LT mixes is significantly less than 17%. This, combined with the fact that the dispersion of the experimental data lies in a range of values wider than 17% (Figure 13 and Figure 14), justifies the impossibility of being able to obtain definitive indications of the trend of the compressive strength over time from Figure 15.

Similar observations and conclusions are valid for the comparison between $q_{90}$ and $q_{180}$—the upper bounds of the compressive strengths at 90 and 180 days—given that Equation (3) returns a percentage increase of 15% between 90 and 180 days of curing:

(7) $$q_{90}=1.312q_{28},$$

(8) $$\frac{q_{180}-q_{90}}{q_{90}}=0.149.$$

### 4.2. Effect of RH Shredding on Stiffness

The trend in the stiffness over time is much richer in information than that of the compressive strength.

**Figure 16:**
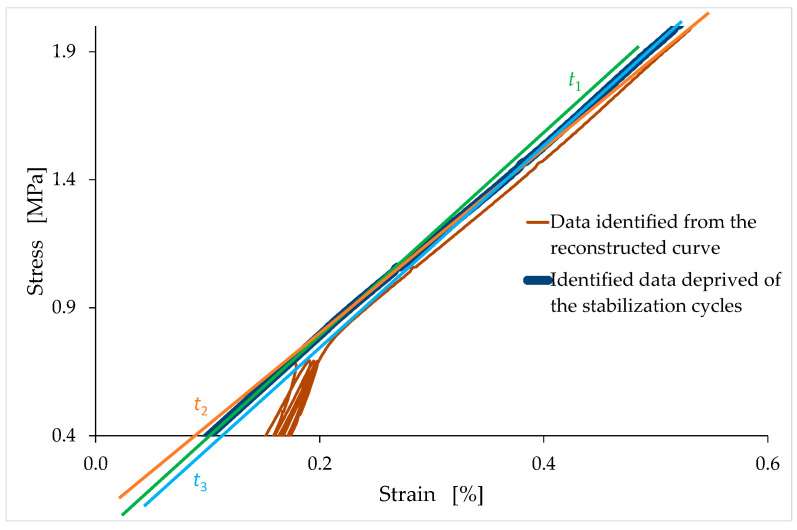
Detail of Figure 9: $t_{1}$ is the tangent line at the end of the linear branch; $t_{2}$ is the tangent line at the inflection point; and $t_{3}$ is the tangent line drawn for a stress value greater than the stress at the inflection point.

The first thing to highlight concerns the behavior of the ascending branch of the stress/strain curve, for both the TQ and LT mixes. In both cases, in fact, after the first linear branch (reconstructed as shown in Section 3.3), the tangent modulus is at first decreasing, and is then subsequently increasing. The tangent lines in Figure 16 show this effect for the LT6 specimen: the slope of the $t_{1}$ line is greater than the slope of the $t_{2}$ line, while the slope of the $t_{2}$ line is less than the slope of the $t_{3}$ line. Therefore, in the central part of the ascending branch, the stress/strain curve has an inflection point, at which the curvature of the stress/strain curve changes signs. This is an anomalous behavior for a brittle material since the tangent modulus in the ascending branch of the stress/strain curve of brittle materials is a monotonic nonincreasing function.

The cause of the continuous decrease in the tangent modulus is the progression of the damage phenomena, which originates in the very first phases of the compressive load test [113]. Obviously, damage phenomena develop in earthen materials in the same way as in all other brittle materials. However, the flex in Figure 16 indicates that a second phenomenon occurs in the ascending branch of the stress/strain curve and counteracts the effect that damage has on the value of the tangent modulus. Since the combined action of the two phenomena causes the material to harden above the flex of Figure 16, the second phenomenon develops less rapidly than the damage in the first part of the stress/strain curve, and more rapidly than the damage after the inflection point. In the remainder of this paper, we will assume that this is the compaction effect, which is due to the closing of the voids caused by the compressive load.

Approaching the peak of the stress/strain curve, the damage phenomena develop more and more rapidly [109], which, again, reverses the ratio between the contributions that the two phenomena make in determining the value of the tangent modulus. This gives rise to a second inflection point—located in the upper part of the ascending branch—with a second inversion in the sign of the curvature. Therefore, as the applied load increases, the ascending branch of the stress/strain curve changes, the first time from being concave (concave downward) to convex (concave upward), and the second time from being convex to concave. In the two concave portions, the damage prevails over the compaction, causing a decrease in the tangent modulus. On the contrary, in the convex portion, the compaction prevails over the damage, causing an increase in the tangent modulus. In the two inflection points, the effects on the tangent modulus of the damage and compaction are equal and opposite, canceling each other out.

**Figure 17:**
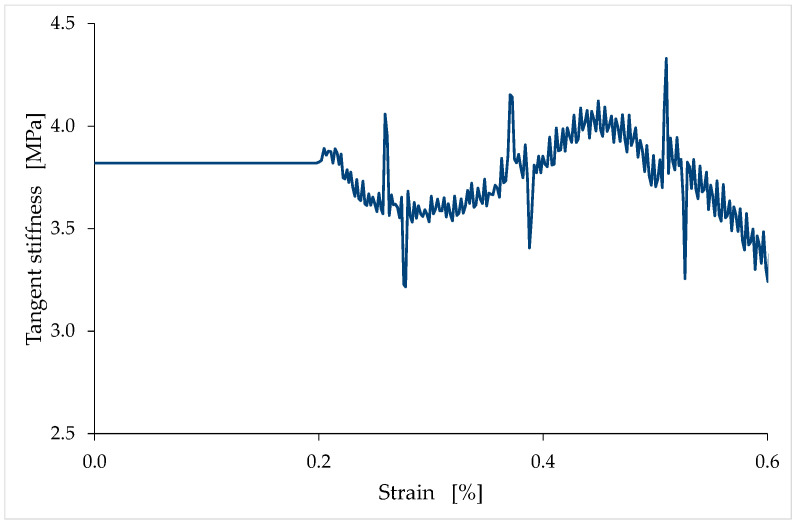
Detail of the slope function for the stress/strain curve (tangent stiffness) of specimen LT6 (n=9): the initial constant value is the slope of the reconstructed straight-line segment.

In Figure 16, the first concave portion begins at the point where the tangent line is $t_{1}$, and ends at the point where the tangent line is $t_{2}$, while the second concave portion begins at the point where the tangent line is $t_{3}$. Between these two concave portions, the curve is convex. To facilitate the identification of the concave and convex portions of Figure 16, Figure 17 shows the slopes of the stress/strain curve (of the LT6 specimen) for the same range of strains as in Figure 16. In the two strain ranges (of Figure 17) where the slope (first derivative) is decreasing (strain values between 0.20% and 0.28%, and greater than 0.44%), the curvature (second derivative) is negative, which means that the stress/strain curve is concave. Conversely, in the strain range (of Figure 17) where the slope is increasing (strain values between 0.28% and 0.44%), the curvature is positive, which means that the stress/strain curve is convex. Finally, the two points of inflection are the two points of Figure 17 where the slope is stationary, which means that the curvature is equal to zero.

Because of the hardening caused by compaction, the maximum tangent modulus in the convex part is greater than the tangent modulus at the origin (Figure 17). Therefore, contrary to what usually happens in brittle materials, the largest of all the tangent moduli along the ascending branch of the stress/strain curve is not the tangent modulus at the origin.

**Figure 18:**
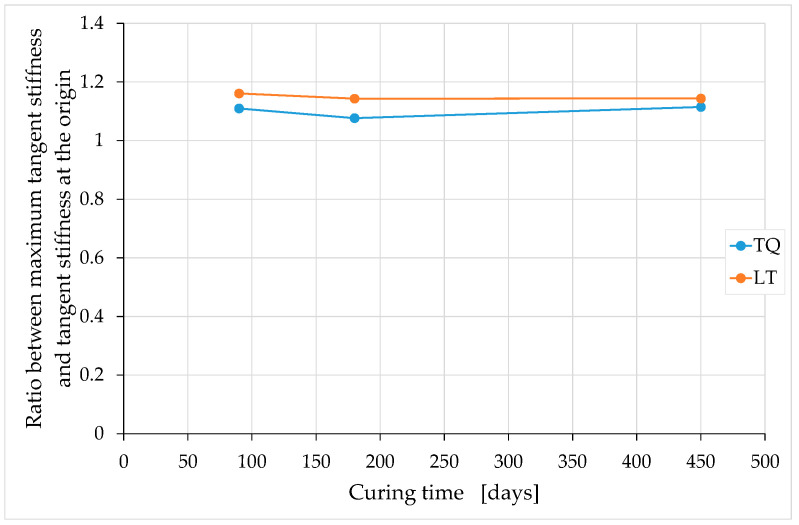
Trend over time of the average values of the ratios between maximum tangent stiffness and tangent stiffness at the origin: TQ and LT mixes.

The ratio between the maximum tangent modulus (maximum tangent stiffness) and the tangent modulus at the origin (tangent stiffness at the origin) is greater for LT specimens than for TQ specimens, regardless of the curing time (Figure 18). This is probably because of the length of the natural fiber, which is greater for the TQ mix than for the LT mix. In fact, a longer natural fiber is more effective in counteracting the movements of the earthen particles under compressive loads, as was already observed by the authors of [68]. Therefore, the compressive load induces less compaction in the TQ specimens than in the LT specimens, which results in a lower hardening effect for the TQ specimens than for the LT specimens.

**Figure 19:**
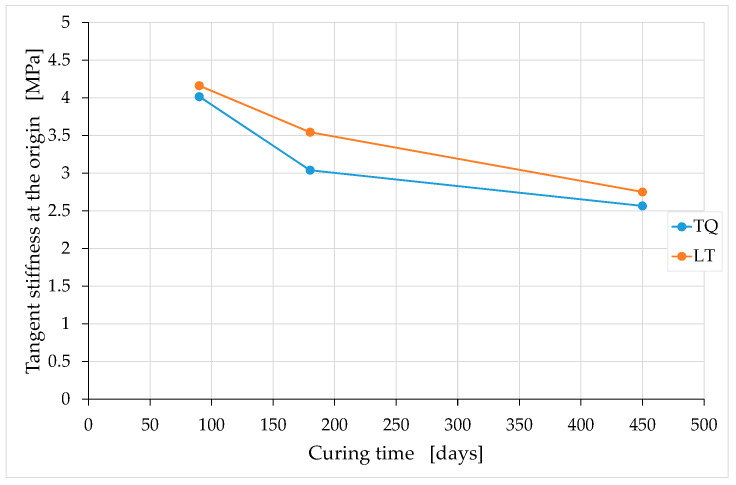
Trend over time of the average values of tangent stiffness at the origin: TQ and LT mixes.

**Figure 20:**
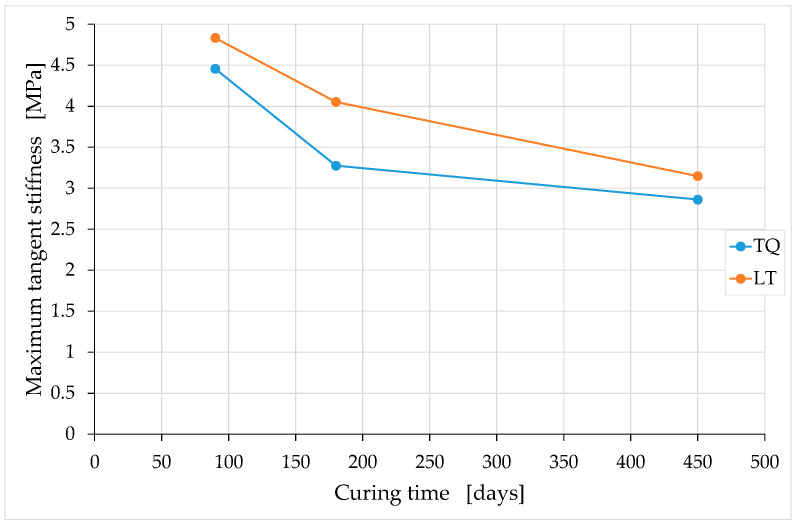
Trend over time of the average values of maximum tangent stiffness: TQ and LT mixes.

In the curing interval analyzed, the ratio between the maximum tangent modulus and the tangent modulus at the origin appears to be almost constant over time (Figure 18). Conversely, both the tangent modulus at the origin and the maximum tangent modulus depend on the curing time, decreasing with it (Figure 19 and Figure 20). In both cases, however, the temporal course of the tangent moduli appears asymptotic, in the sense that the tangent moduli converge towards constant values at infinite time.

The decrease over time in the tangent moduli indicates that the deformability under compressive loads of the earthen mixtures increases over time. To the best of the knowledge of the authors, this is the first time that an experimental campaign on earthen mixtures highlights the decrease over time in tangent moduli. This is actually the first experimental campaign conducted over a sufficiently long period to highlight how the mechanical properties of the earthen mixtures vary over time, particularly after long curing times.

Figure 19 and Figure 20 also show that the two tangent moduli of the LT mix are greater than the two tangent moduli of the TQ mix at any time of curing. In other words, the LT specimens are stiffer than the TQ specimens are. This confirms the idea that inspired the experimental campaign (Section 2), according to which the shredding of the RH would have a greater biocementing effect on the mixture, as it favors the formation of Si–O–Si bonds. In fact, a greater number of Si–O–Si bonds in the mixture counteracts the deformability of the material, resulting in greater stiffness. In light of the observations made for the compressive strength (Section 4.1), it is likely that the preventive premixing of the lime with the RH can enhance the biocementing effect of the shredding, leading to even greater increases in stiffness than those observed in Figure 19 and Figure 20.

**Figure 21:**
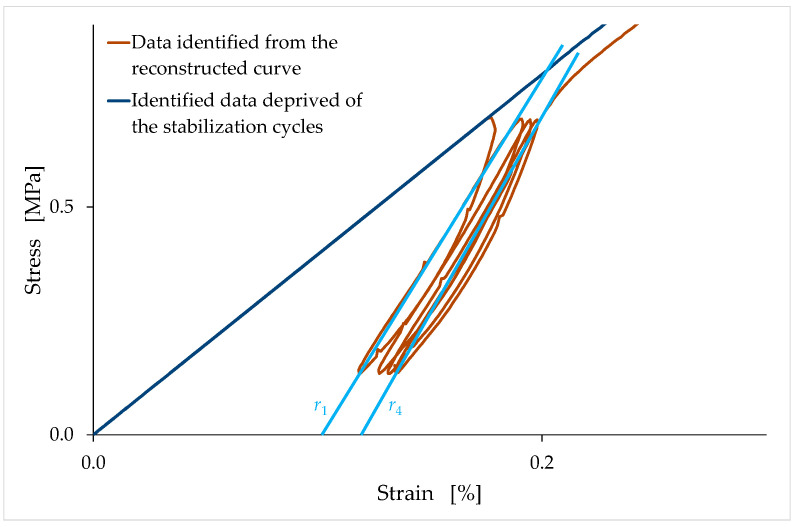
Evaluation of the average slope of unloading–reloading in a cycle as the slope of the straight line that joins the lower point of the cycle with the intersection point of the cycle.

To complete the study on how the mechanical properties of earthen mixtures vary over time, it is advisable to analyze how the slopes of the stabilization cycles vary over time. Being, in fact, unloading–reloading cycles, the stabilization cycles provide information on the elastic and plastic components of the strain at the unloading stress value. Since the stabilization cycles are hysteretic, it is necessary to define their average slope conventionally: the average slope of a stabilization cycle is the slope of the straight line that joins the lower point of the cycle with the intersection point of the cycle. In Figure 21, the straight line $r_{1}$ provides the slope of the first stabilization cycle, and the straight line $r_{4}$ provides the slope of the fourth stabilization cycle. As the stabilization process proceeds, the widths of the hysteretic cycles decrease, and the average slope of the cycles changes slightly (Figure 21).

The conventional slope of the unloading–reloading is the slope of the straight line that approximates the last stabilization cycle (line $r_{4}$ in Figure 21). Being a slope in the stress/strain plane, this value represents a stiffness. In natural brittle materials, the slope of a stabilization cycle is greater than the tangent stiffness at the origin.

It is worth noting that the slope of $r_{4}$ in Figure 21 is greater than the slope of $r_{1}$, and, more generally, the average unloading–reloading slope in Figure 21 increases as the stabilization proceeds. This is in contrast to what happens in brittle materials, as, usually, the average slope of a stabilization cycle decreases as the stabilization proceeds. This anomalous result is a further effect of the compaction that occurs—in the latter case, under cyclic load—because of the partial closure of the voids induced by compression. We could say that the increase in the slopes of Figure 21 is an indirect confirmation of the effectiveness of the mechanical tamping, developed on an empirical basis as a means to stabilize the earthen mixtures in the rammed earth technique (Section 1.2).

**Figure 22:**
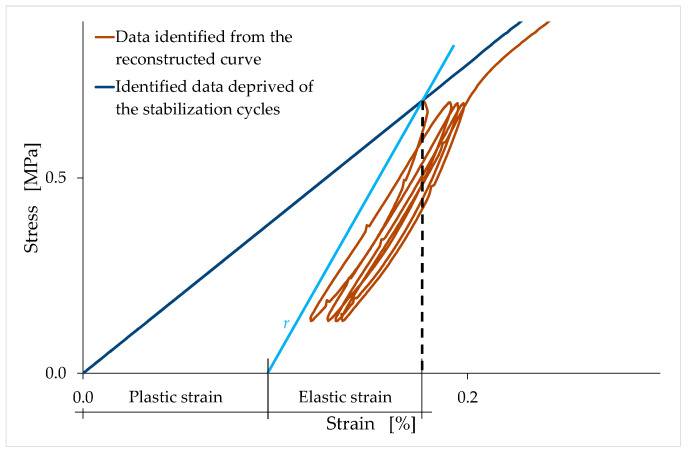
The two components of the strain at the stabilization stress value: the elastic strain is the strain recovered at the end of an ideal straight path of unloading, carried out for the strain value under consideration (the straight line, r, is parallel to the straight line $r_{4}$ in Figure 21); the plastic strain is the permanent strain after unloading.

By convention, the unloading carried out for the stabilization stress value, in the curve without stabilization cycles (Section 3.3), follows an ideal straight path, with a slope equal to the unloading–reloading slope identified as above (line r in Figure 22). This straight line provides the elastic and plastic components of the strain for the stress value at unloading: the elastic and plastic strains are, respectively, the recovered and the permanent strains after the complete removal of the external load (Figure 22).

**Figure 23:**
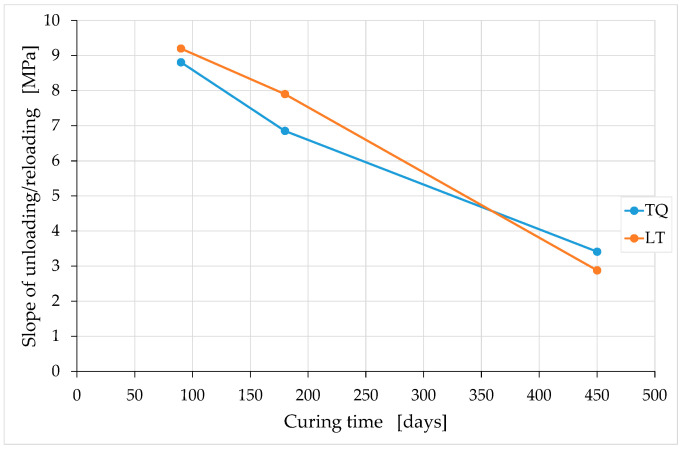
Trend over time of the average values of the unloading–reloading slope (TQ and LT mixes): the unloading–reloading slope is the slope of the last stabilization cycle.

Figure 23 shows the average values of the slopes of unloading–reloading for the groups of three specimens with the same curing times. As with the tangent moduli, these values depend on the curing time, but the behavior is not asymptotic over time.

**Figure 24:**
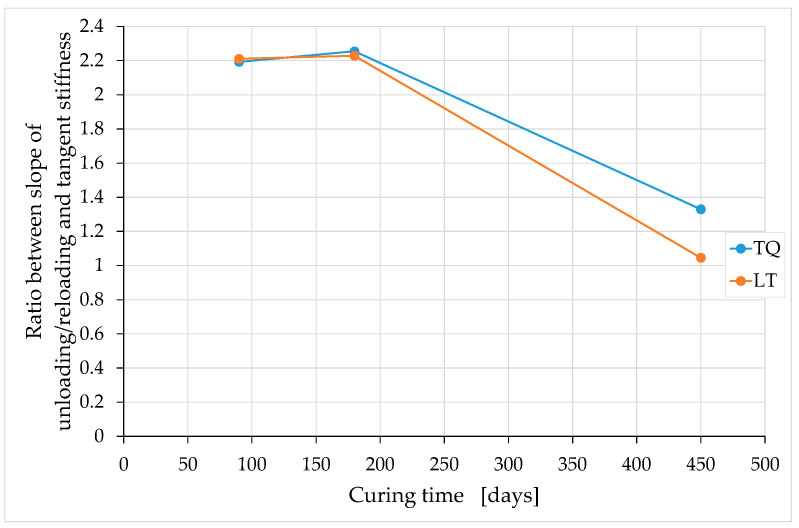
Trend over time of the average values of the ratio between unloading–reloading slope and tangent stiffness at the origin: TQ and LT mixes.

Of particular interest is the relationship over time between the unloading–reloading slope (which is a stiffness) and the tangent stiffness at the origin. As previously mentioned, in natural brittle materials, including earthen mixtures, the ratio between these two values is greater than one. The TQ and LT mixtures, however, behave abnormally (for a natural material) over long curing periods (Figure 24).

From a mathematical point of view, the trend in Figure 24 derives from the fact that, while the tangent moduli at the origin tend towards constant values at infinite time (Figure 19 and Figure 20), the unloading–reloading slopes decrease over time with an almost linear law (Figure 23). From a physical point of view, Figure 24 indicates that the elastic recovery at unloading increases significantly after 180 days of curing, for both mixtures. In fact, a decrease (towards the unit value) in the ratio between the two slopes (stiffnesses) leads to an increase in the percentage deformation recovered after the complete removal of the external load, as well as to a decrease in the percentage of plastic deformation. In the ideal case of a ratio equal to unity, all the deformation accumulated before unloading is recoverable, and there is no permanent deformation after the complete removal of the external load. This means that the specimen will revert to its original shape, regardless of the amount of deformation accumulated prior to unloading (perfectly elastic behavior).

The anomalous behavior of the TQ and LT mixes is, precisely, the sharp decrease in the ratio between the stiffnesses after 180 days of curing, which usually does not occur spontaneously in a natural material. It is, in fact, an effect similar to that produced in natural rubber by vulcanization, which is a chemical process used in the processing of rubber that causes natural rubber to lose its essentially plastic characteristics and acquire those of an essentially elastic, and not very swellable, material (if kept in contact with organic solvents). In a broader sense, the term “vulcanization” includes any chemical reaction that produces similar effects, not only on natural rubber, but also on synthetic rubber or any other material. Apart from the fact that it is a natural process and not a chemically induced one, we could, therefore, say that curing has a “vulcanizing” effect on the TQ and LT mixes. In particular, it is possible that the bonds formed thanks to the presence of silica in the RH have an effect similar to that of the cross links between polymer chains induced by vulcanization. In the specific case of the LT mix, a curing period of 450 days causes the specimens to lose their plastic behavior almost completely, up to a stress value equal to the stabilization stress. It is worth noting that the dependence of the ratio between the slopes on the curing time is only apparently linear between 180 and 450 curing days (Figure 24), since we only have initial and final data in this range. Because of the relevance of this relationship in defining the mechanical behavior of the material in the long term, in the future, it will be appropriate to collect more data in this time interval.

Because of the lack of data between 180 and 450 curing days, it is not possible to formulate reliable hypotheses on the trend of the ratio between the stiffnesses beyond 450 curing days. At first glance, however, Figure 24 seems to indicate that the stiffness ratio can assume values below unity beyond 450 curing days, especially for the LT mix. This would mean that the strain recovered at unloading is even greater than the strain at the time of unloading, a very anomalous behavior for a natural material.

Figure 24 also shows that the increase in the elastic recovery after 180 days of curing is greater for the LT mix than for the TQ mix. The greater reduction in the plastic component in the LT mix is due to the greater number of Si–O–Si bonds induced by the shredding of the RH, since the shredding generates a greater contact surface between the RH and the matrix. In fact, the Si–O–Si bonds hinder the plastic flow, as well as increase the stiffness.

## 5. Conclusions and Future Developments

This paper presents the results of uniaxial compression tests performed on two different earthen mixtures. The two mixtures differed only in the particle size of the natural fiber, which was unaltered rice husk for the first mixture (TQ mix), and shredded rice husk for the second mixture (LT mix). The mix designs of both mixtures make them suitable for use by extrusion in the 3D printing of earthen buildings. The attention of this experimental campaign did not focus on the (already investigated) basic criteria that guarantee good printability properties (extrudability, buildability, and workability with time), but rather on the variation over time of the mechanical properties under compression load. To this end, we performed compression tests at 90, 180, and 450 days of curing.

The experimental results highlight the anomalous behaviors over time of the two earthen mixtures, particularly for long curing times. Some of these behaviors are clearly recognizable after a curing time of 450 days. However, the experimental results, on the whole, indicate that it is necessary to carry out analyses on longer curing times in order to draw definitive conclusions on the trend over time of the mechanical properties of the earthen mixtures.

The most surprising results of this experimental campaign are two: the stiffness decreases over time with an asymptotic behavior, and a vulcanization effect occurs spontaneously with aging. For a correct understanding of the mechanical behavior of earthen materials, it is necessary to analyze these two results together. In fact, the second one (spontaneous vulcanization) proved to be able to cancel most of the negative effects of the first one (decrease in stiffness). The prevalence of the vulcanization effect on the decrease in stiffness is particularly evident for the LT specimens, in which it allows for the recovery (at unloading) of almost all the strains reached by the specimens at the time of unloading, if subjected to a compression load test after a curing time of 450 days, and if unloaded at the stabilization load value. To be precise, the trend over time of the two phenomena would seem to indicate that the recovery of strain at unloading could be even greater than the strain at the time of unloading, for periods of curing exceeding 450 days, and for an unloading load equal to the stabilization load. In the case of the confirmation of this trend, it would mean that the earthen mixtures are very suitable for use in repeated loading conditions, being able—in an advanced stage of curing—to recover most of the plastic strains accumulated in the first load cycles. For a better understanding of this behavior, it is advisable to carry out uniaxial compression tests with unloading–reloading cycles at regular displacement intervals (in addition to the stabilization cycles), also taking into account curing periods longer than 450 days.

A further result of the experimentation concerns the higher stiffness values obtained for the LT specimens compared to those obtained for the TQ specimens. This is actually the expected result that motivated the experimentation itself. The higher stiffnesses of the LT specimens find an explanation in the greater contact surface generated between the rice husk and the matrix through the shredding of the rice husk. In fact, those responsible for the increase in stiffness are the Si–O–Si bonds that can form on the contact surface, thanks to the reaction between the lime and the silica contained in the rice husk. Since a larger contact surface generates a greater number of Si–O–Si bonds, the larger contact surface is, therefore, also responsible for the higher stiffnesses of the LT specimens compared to the TQ specimens.

The analysis of the experimental data led to the conclusion that, in order to obtain the greatest possible number of Si–O–Si bonds, with all other conditions being equal, it is advisable to premix the lime and the rice husk before adding them to the other components of the mixture. This could allow us to achieve that increase over time, in terms of compressive strength and stiffness, which we did not observe in these tests. According to the scientific literature, it is probable that the failure to increase the compressive strength over time also depends on the curing time interval considered. As for the stiffness, it is probable that the asymptotic decreasing trend is precisely a characteristic of the soil, and that the quantity of the Si–O–Si bonds was not sufficient to reverse this trend. This latter aspect also deserves further study.

Finally, the premixing of the lime and rice husk would also have a positive effect on the durability. In fact, by ensuring that all the rice husk reacts with the lime, the premixing would favor the mineralization of all the rice husk, preserving it from bacteria, molds, insects, rodents, and fire. This is a very important aspect, especially with regard to the preservation from the mold attacks of the LT mix. In fact, since this mixture has shown some problems of workability, in order to make it suitable for 3D printing, it may be necessary to increase its water content, which notoriously favors the formation of molds.

## Figures and Tables

**Table 1:** Percentage compositions by weight of the TQ and LT mixes.

Component	TQ Mix	LT Mix
Soil	70.42%	70.42%
Lime-based binder	4.70%	4.70%
Hydraulic lime	4.69%	4.69%
Unaltered rice husk	1.41%	/
Shredded rice husk	/	1.41%
Silica sand	18.78%	18.78%

**Table 2:** Sieve analysis of the soil sample collected on site (EN 17892-4:2005).

Opening Diameter (mm)	Cumulative Retained (%)
8	0
4	0.1
2	0.5
1	1.0
0.4	2.0
0.075	7.1
0.001	100

**Table 3:** List of the specimens of the experimental program (after the elimination of the discarded specimens).

Specimen Label	Type of Mixture	Curing Days
TQ1	TQ mix	90
TQ2	TQ mix	90
TQ3	TQ mix	90
TQ4	TQ mix	180
TQ5	TQ mix	180
TQ6	TQ mix	180
TQ10	TQ mix	450
TQ11	TQ mix	450
TQ12	TQ mix	450
LT1	LT mix	90
LT2	LT mix	90
LT3	LT mix	90
LT4	LT mix	180
LT5	LT mix	180
LT6	LT mix	180
LT7	LT mix	450
LT8	LT mix	450
LT9	LT mix	450

**Table 4:** Densities in the dry state of the nine specimens made with the TQ mix, and the nine specimens made with the LT mix (for the meaning of symbols, see Equation (1)).

Mixture	$\rho_{min}$ [kg/m^3^]	$\rho_{max}$ [kg/m^3^]	$\bar{\rho }$ [kg/m^3^]	ξ [%]
TQ mix	1418.116	1495.728	1446.360	3.4
LT mix	1422.778	1494.151	1446.687	3.3

## Data Availability

Not applicable.

## Supplementary Materials

The following are available online at https://www.mdpi.com/article/10.3390/ma15030743/s1. Figure S1: The BigDelta 3D printer made by WASP; Figure S2: The Crane WASP: a collaborative 3D-printing system targeted to end-use production of functional buildings; Figure S3: The Gaia House by WASP: a 3D-printed prototype built with biodegradable materials; Figure S4: Filling of Gaia’s honeycomb structure with rice husk; Figure S5: Floorplan of Gaia; Figure S6: Four of the eight wooden pillars that sustain Gaia’s roof; Figure S7: TECLA’s honeycomb structure; Figure S8: The two domes of TECLA in an advanced stage of completion; Figure S9: Some of TECLA’s interior furnishings; Figure S10: The lime cycle; Figure S11: Flowchart of WASP’s 3D-printing process for earthen construction; Video S1: Unveiling the new WASP house 3D printer|Event 6–7 October 2018; Video S2: Gaia|3D-printed earth house with Crane WASP|Presentation Video; Video S3: Ecosustainable 3D-printed house—TECLA; Table S1: A review of scientific articles on earthen construction, published in the period 1948–2019; Table S2: The eight categories of soil biostabilization.
