# Supplementary material for: Rice-Husk Shredding as a Means of Increasing the Long-Term Mechanical Properties of Earthen Mixtures for 3D Printing
Source: Materials (Basel). 2022 Jan 19;15(3):743. doi: 10.3390/ma15030743 (PMC8836500; doi:10.3390/ma15030743)
Supplement: Supplementary file 1 [file materials-15-00743-s001.zip › materials-1543421-Supplementary_material-Final/materials-1543421-Supplementary_material-Final.pdf]

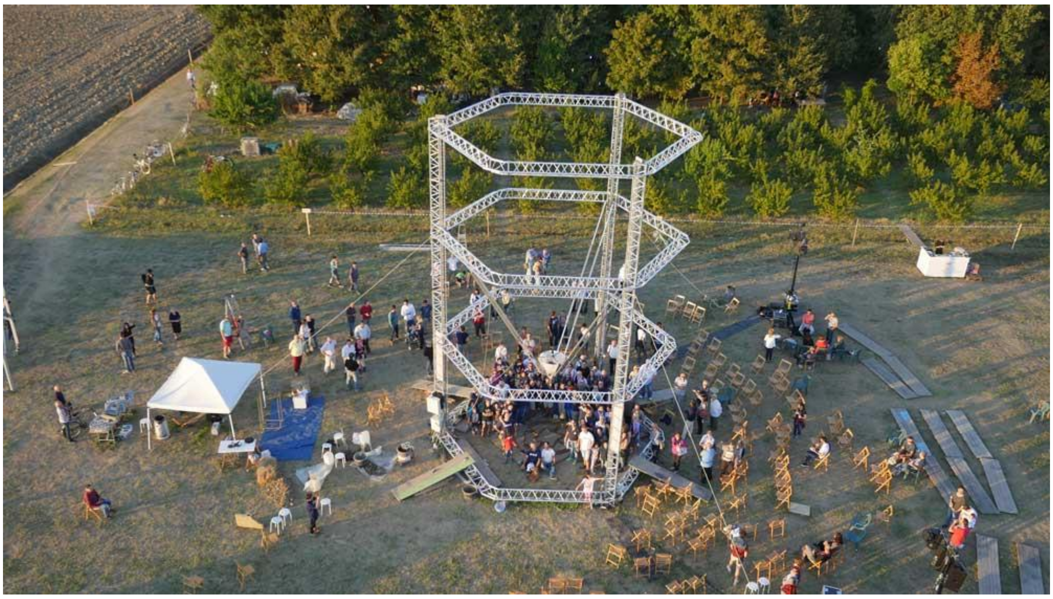

Figure S1. The BigDelta 3D printer made by WASP [source WASP].

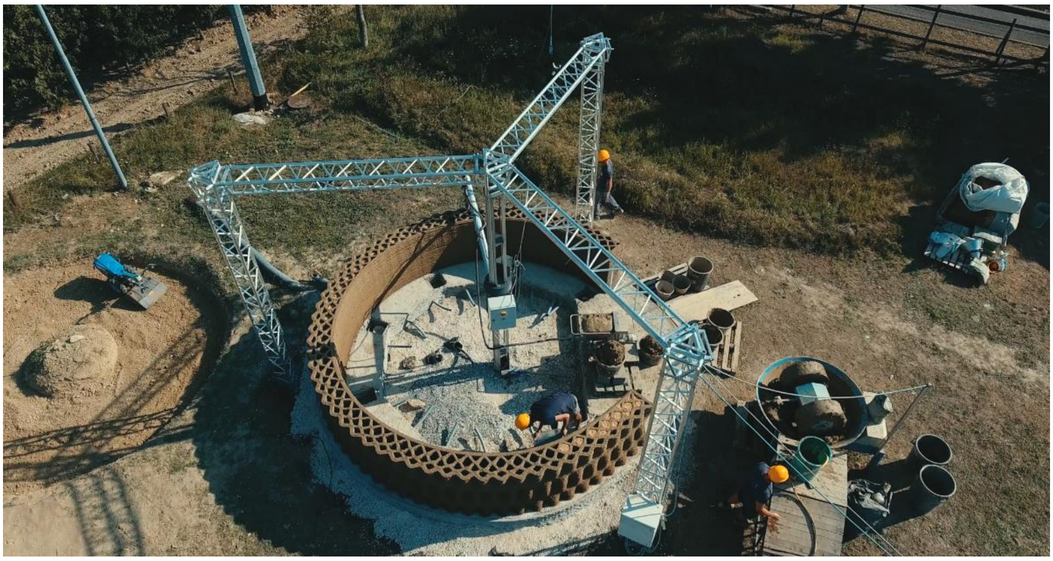

Figure S2. The Crane WASP: a collaborative 3D-printing system targeted to end-use production of functional buildings [source WASP].

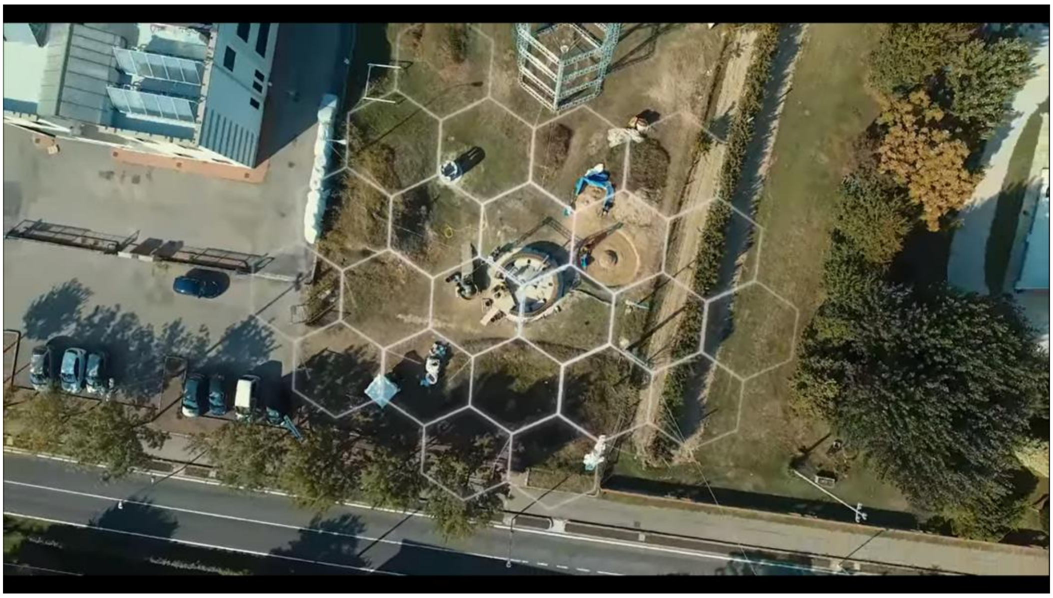

Video S1. Unveiling the new WASP house 3D printer | Event 6-7 October 2018; https://www.youtube.com/watch?app=desktop&v=KS1mb8QVE-E&list=PLKS-fMq7r3YolyGxCU8xhfNLVcLH5KMhxT&t=0s&index=7 [source WASP].

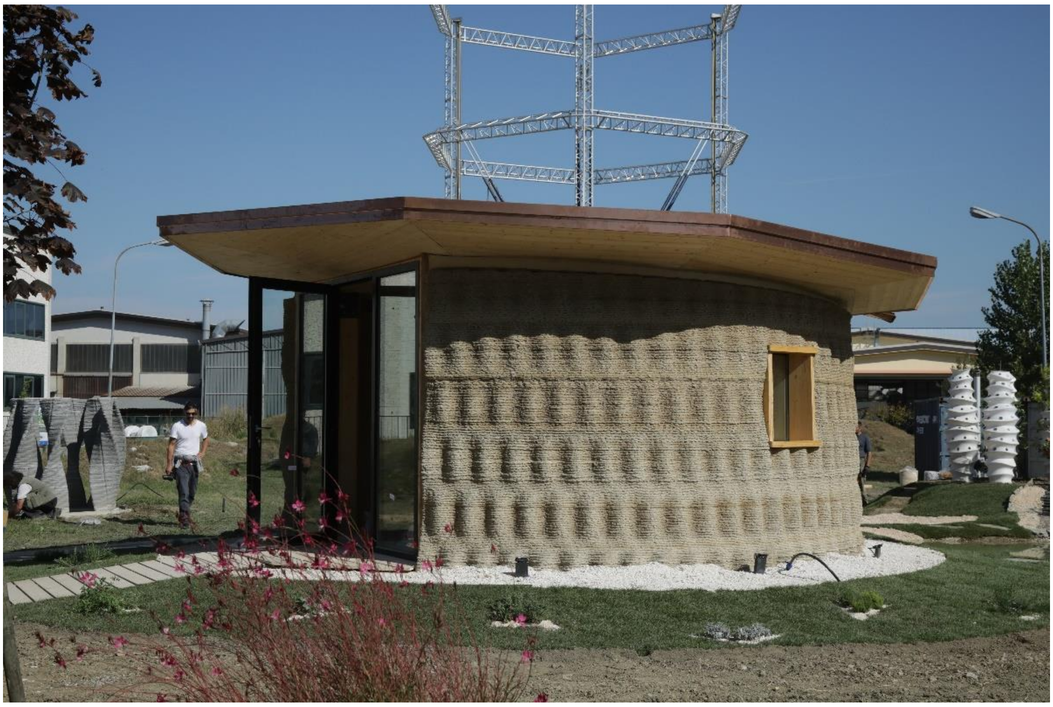

Figure S3. The Gaia House by WASP: a 3D-printed prototype built with biodegradable materials [source WASP].

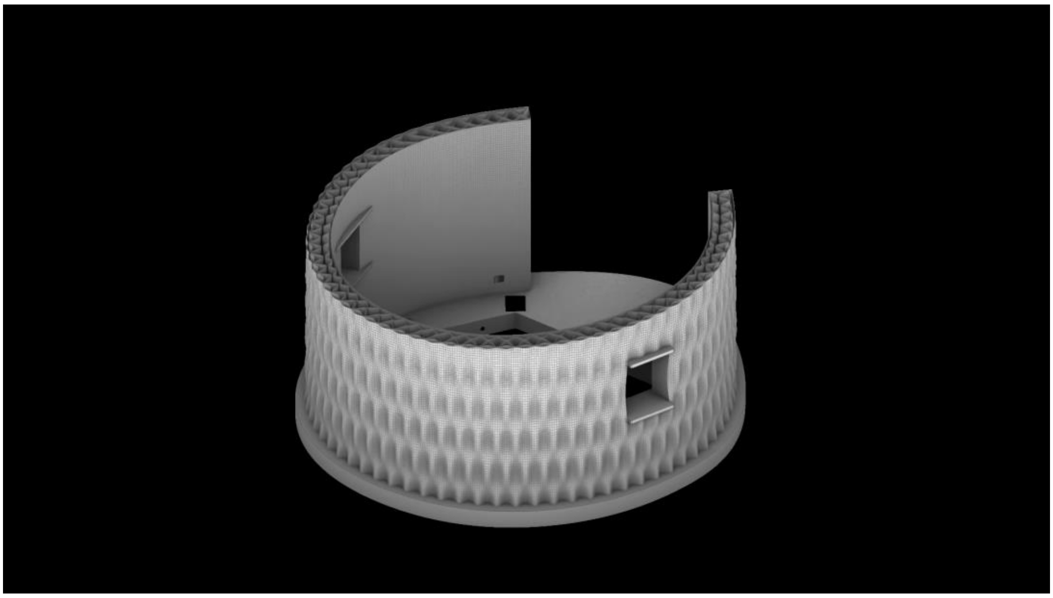

Video S2. Gaia | 3D-printed earth house with Crane WASP | Presentation Video; https://www.youtube.com/watch?app=desktop&v=KPaOCWrZJ94 [source WASP].

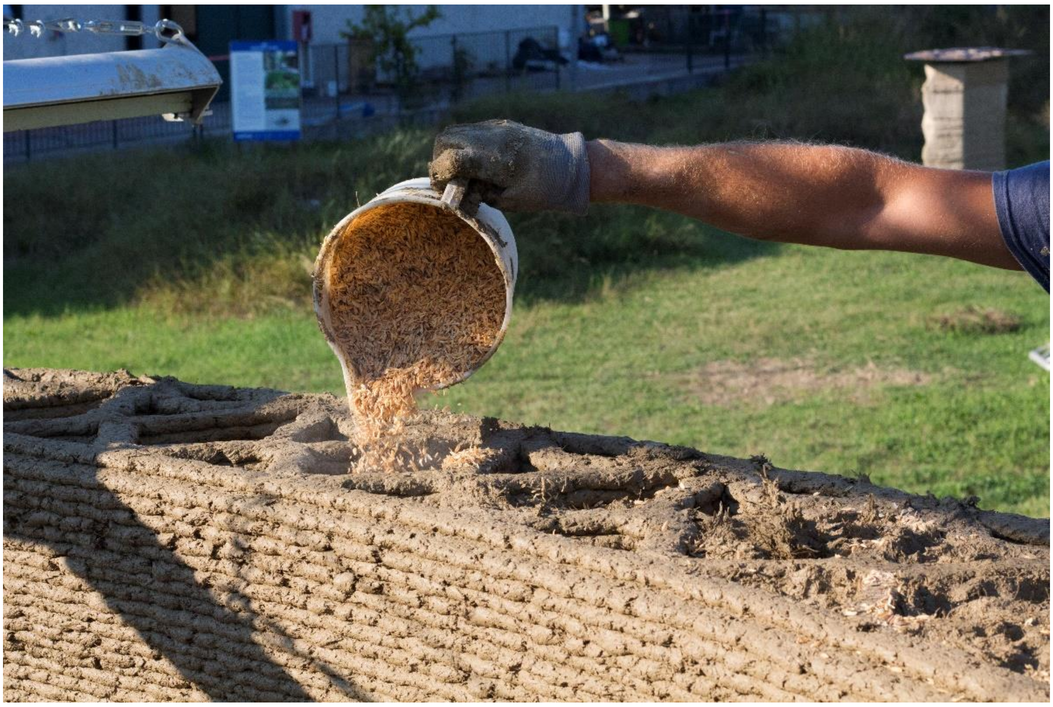

Figure S4. Filling of Gaia's honeycomb structure with rice husk [source WASP].

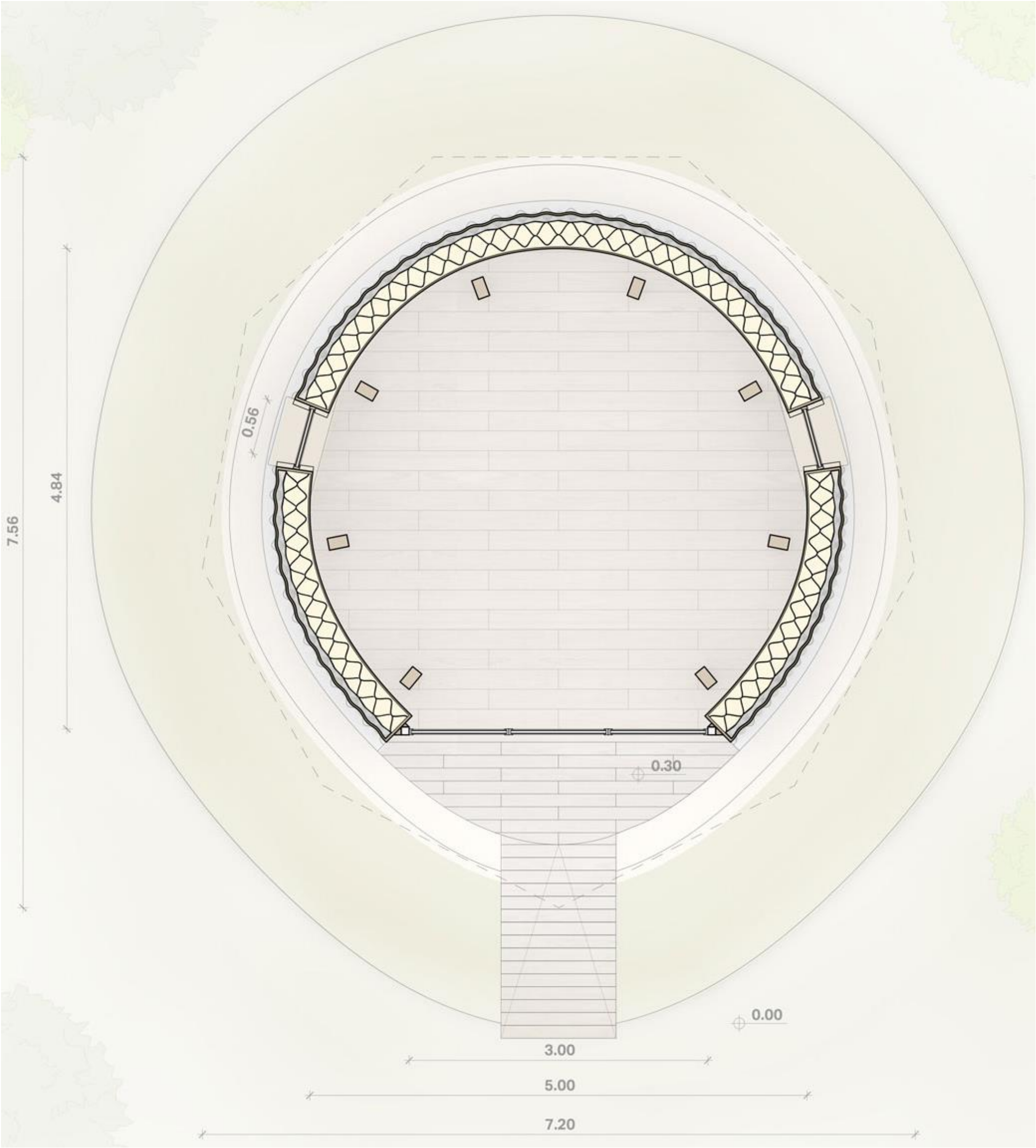

Figure S5. Floorplan of Gaia [source WASP].

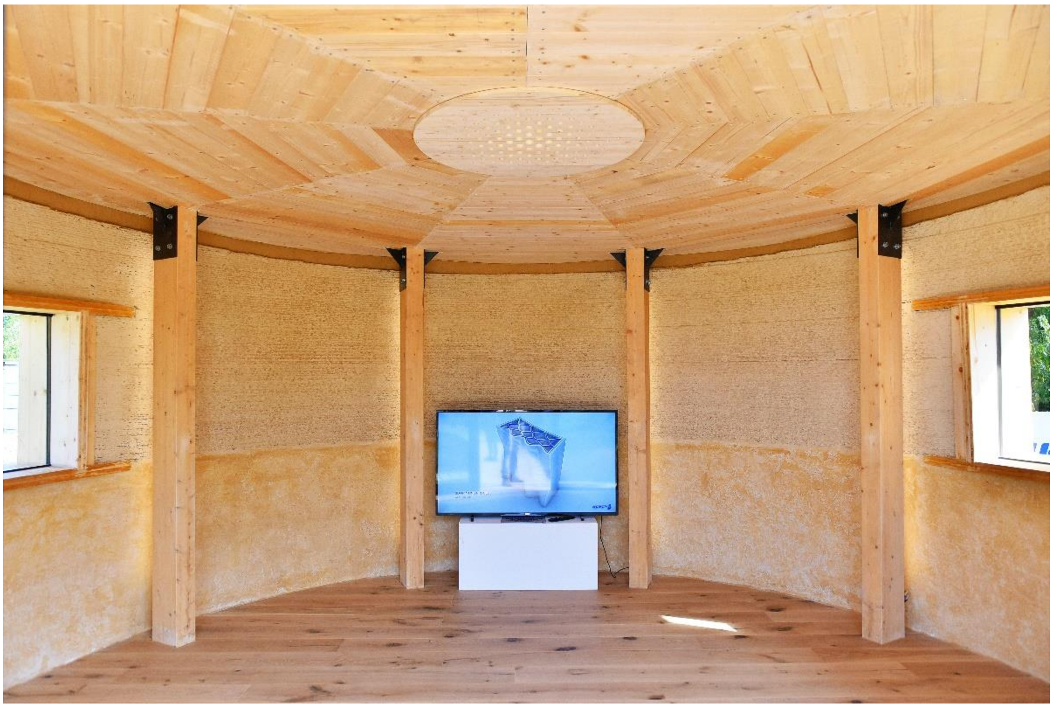

Figure S6. Four of the eight wooden pillars that sustain Gaia's roof [source WASP].

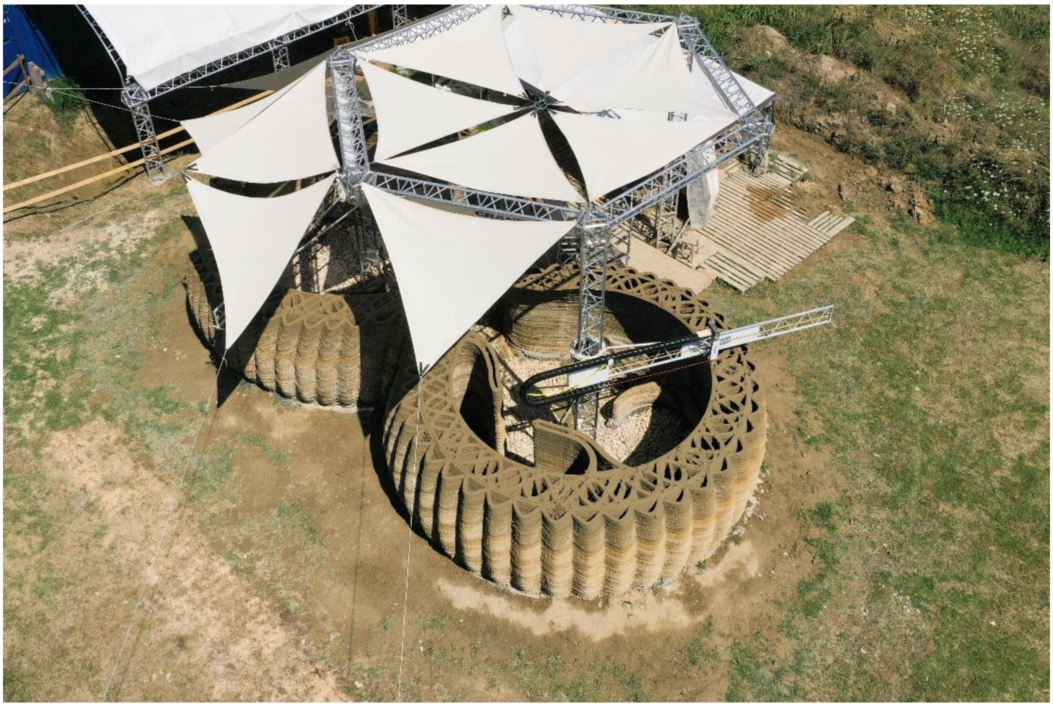

Figure S7. TECLA's honeycomb structure [source WASP].TECLA

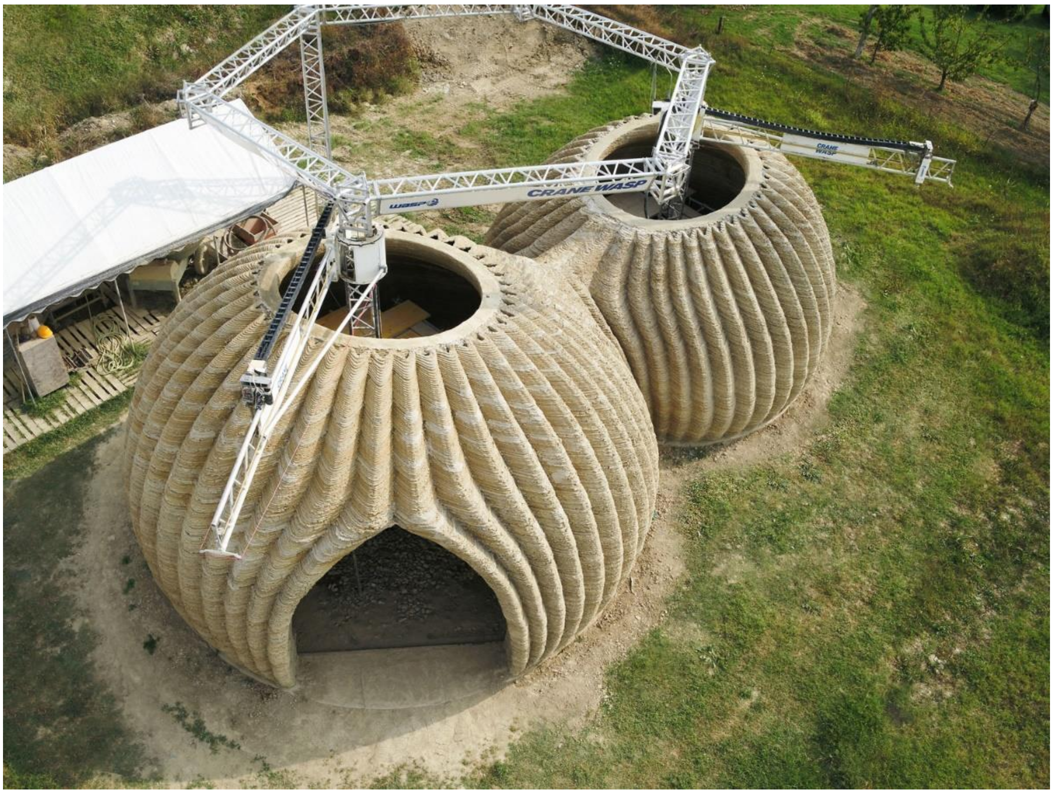

Figure S8. The two domes of TECLA in an advanced stage of completion [source WASP].

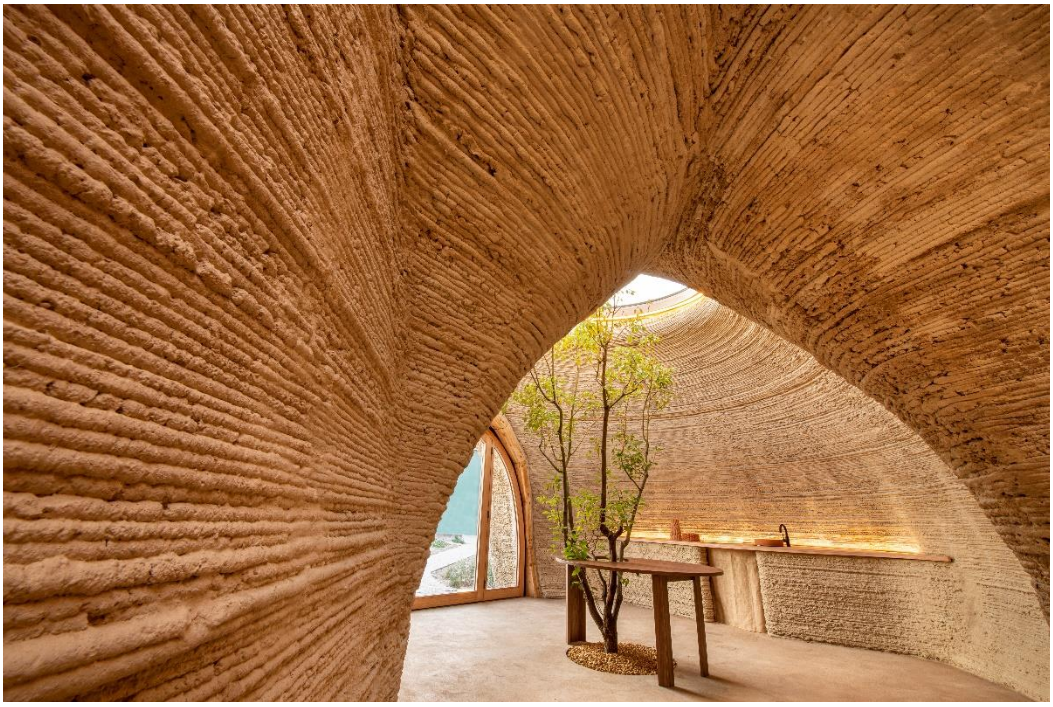

Figure S9. Some of TECLA's interior furnishings [photo taken by Iago Corazza].

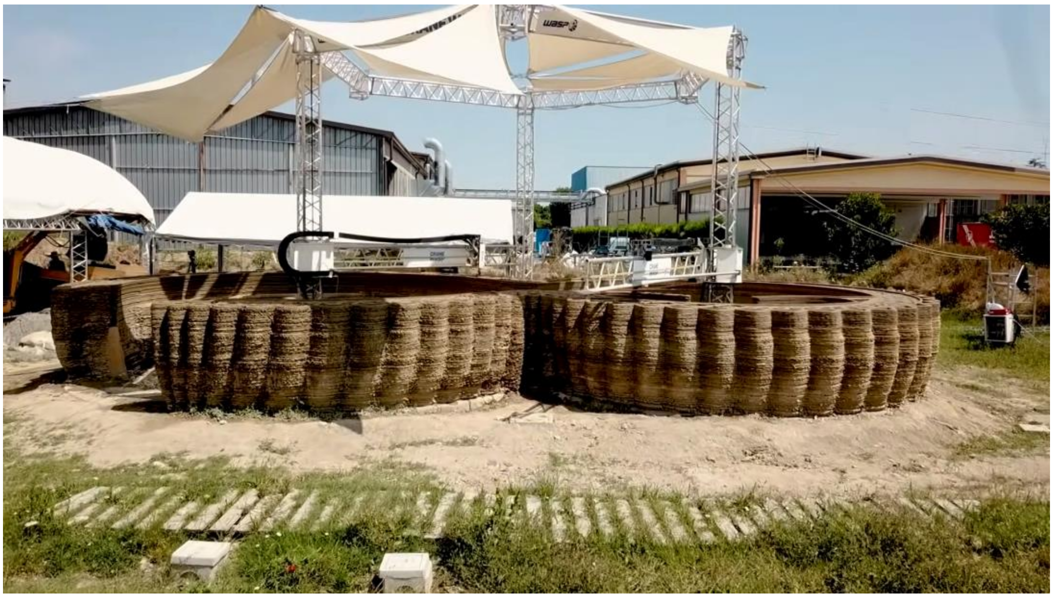

Video S3. Eco-sustainable 3D-printed house—TECLA; https://www.youtube.com/watch?app=desktop&v=w9sXqxccRPM&feature=youtu.be [source WASP].

Table S1. A review of scientific articles on earthen construction, published in the period 1948–2019.

Reference(s)	Topic(s) of the paper(s)
[10,20–29]	How factors such as particle size distributions, gravel content, sand content, silt and clay content (content of fine fractions), water content, stabilizer content, density and compaction define the strength characteristics of an earthen material.
[30]	Plasticity and shrinkage properties of the earthen construction material: how the chemical composition of clay particles allows the particles to interact with the environment to attract moisture to their surfaces, modifying the hygroscopic behavior of the earthen material.
[31]	Plasticity and shrinkage properties of the earthen construction material: how the drying/wetting cycles due to environmental conditions lead to the reduction of strength and stiffness as a result of the shrinkage/swelling of the clays within the earthen material.
[21]	Plasticity and shrinkage properties of the earthen construction material: range of soil plasticity for unstabilized rammed earth and compressed earth blocks.
[21,32–34]	Plasticity and shrinkage properties of the earthen construction material: range of soil plasticity for concrete stabilized earthen materials in rammed earth.
[23,35,36]	Plasticity and shrinkage properties of the earthen construction material: range of soil plasticity for concrete stabilized earthen materials in compressed earth blocks.
[21,37–41]	Grain size recommendations: unstabilized earthen materials for use in rammed earth.
[21,38–40]	Grain size recommendations: unstabilized earthen materials for use in compressed earth blocks.
[32,34,42,43]	Grain size recommendations: stabilized earthen materials for use in rammed earth.
[11,23,35,36,41,44–48]	Grain size recommendations: stabilized earthen materials for use in compressed earth blocks.
[49,50]	Classification of soils suitable for rammed earth construction, based on soil grain size, plasticity and shrinkage properties.
[11,21,35,45,46,51,52]	Role of different stabilizers in the preparation of Compressed Stabilized Earth Blocks (CSEBs) with improved strength and durability performance.
[21,35,36,46,53–55]	Optimal stabilizer content for CSEBs.
[56,57]	Durability: rain-induced erosion and deterioration from capillary rising.
[23]	Durability: effect of different plasticity properties on cement stabilized earth blocks.
[58]	Durability: performance of stabilized (with cement and/or lime) compressed blocks under both laboratory and climatic conditions exposure.

[59]	Durability: effect of exposure to natural atmospheric agents for 20 years on rammed earth walls.
[60]	Durability: strength and durability properties of 32-year-old cement stabilized rammed earth (CSRE) and 28-day-old rammed earth specimens.
[61]	Durability: effect of stabilizers other than cement on the mechanical and durability properties of hyper-compacted earthen materials.
[30,62–65]	How the hygroscopic behavior of earthen construction materials determines indoor air quality and humidity levels.

Table S2. The eight categories of soil biostabilization.

Name	Description
Bio-aggregation	Increasing the effective size of the soil particles to reduce soil erosion and dust emission.
Bio-crusting	Formation of a crust on the soil surface to resist wind and water erosions and to reduce dust emission and water erosion.
Bio-coating	Formation of a layer on the soil surface in order to improve colonization, aesthetics, or resistance to corrosion.
Bio-clogging	Filling the soil pores or rock cracks to reduce the permeability of the material.
Bio-cementation	Binding the soil particles to increase shear strength.
Bio-desaturation	Production of biogas bubbles in situ to reduce saturation and liquefaction potential of the soil.
Bio-encapsulation	Increasing the strength by encapsulating certain soil particles, such as soft clay, loose sand, quick sand and muck soil.
Bio-remediation	Bio-degradation of soil pollutants in contaminated soils.

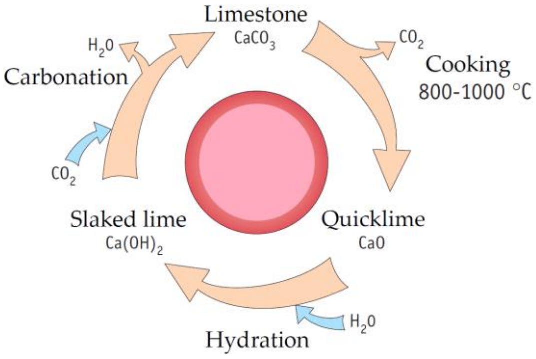

Figure S10. The lime cycle: (**1**) cooking calcium carbonate ($CaCO_3$) at 800-1000 °C (process known as calcination) produces quicklime, namely calcium oxide (CaO), with the release of carbon dioxide (CO_2); (**2**) the reaction of quicklime with water (hydration) causes a violent release of heat and the transformation of calcium oxide into calcium hydroxide ($Ca(OH)_2$), called hydrated lime or slaked lime, suitable for use in construction; (**3**) the prolonged contact of the slaked lime with the carbon dioxide contained in the air triggers a carbonation reaction that transforms the calcium hydroxide into calcite (calcium carbonate), with the release of water.

Architectural design:
definition of the volumes and geometry of the building to be built

↓

Preparation of the 3D model of the building to be built

↓

Writing the G-code

↓

Assembly of the 3D printer on the construction site:
in the basic version, the work of three people is required for one hour

↓

Powering the 3D printer:
in the basic version, a few meters of solar panels are enough to power the 3D printer

↓

Starting the 3D printer

↓

Soil supply:
excavation of all the necessary soil from the construction site

↓

Soil selection:
sieving of the soil using a screening bucket (grain size 0-6 mm), to remove stones and other impurities

↓

Soil qualification:

- Water content (ASTM D216-10)
- Specific gravity of solid soils (ASTM D854-10)
- Density (EN 13286-2: 2005)
- Grain size analysis: sieve distribution (EN 17892-4:2005)
- Organic matter content (ASTM D2974-07a:2012)
- HRB/UNI EN ISO 14688-1:2018 soil classification

↓

Soil stabilization:
mixing of the soil with the RH-lime biocomposite, in the muller

↓

Preparation of the earthen mixture:
mixing of the stabilized soil with the other components of the mix-design and the mixing water, in the muller

↓

Transfer of the mixture from the muller to the feed pump of the 3D printer

↓

3D printing of the earthen building

Figure S11. Flowchart of WASP's 3D-printing process for earthen construction: the figure shows two different mixing phases in the muller, the first aimed at stabilizing the soil and the second aimed at kneading the mixture. In reality, (as explained in the main test) up to now WSAP has made use of only one mixing phase, which consists in simultaneously inserting all the components of the mix-design into the muller, together with the mixing water. The opportunity to carry out the mixing in two different moments is one of the results that emerged from the present paper, as this seems to favor the bio-cementing action of the bio-composite.

References

1. Kothman, I.; Faber, N. How 3D printing technology changes the rules of the game: Insights from the construction sector. *J. Manuf. Technol. Manag.* **2016**, *27*, 932–943.

2. Tay, Y.W.D.; Panda, B.; Paul, S.C.; Noor Mohamed, N.A.; Tan, M.J.; Leong, K.F. 3D printing trends in building and construction industry: A review. *Virtual Phys. Prototyp.* **2017**, *12*, 261–276.
3. Alhumayani, H.; Gomaa, M.; Soebarto, V.; Jabi, W. Environmental assessment of large-scale 3D printing in construction: A comparative study between cob and concrete. *J. Clean. Prod.* **2020**, *270*, 122463. https://doi.org/10.1016/j.jclepro.2020.122463.
4. Veliz Reyes, A.; Gomaa, M.; Chatzivasileiadi, A.; Jabi, W.; Wardhana, N.M. Computing craft: Early stage development of a robotically-supported 3D printing system for cob structures. In Proceedings of the 36th eCAADe Conference, Lodz University of Technology, Lodz, Poland, 19–21 September 2018; Kepczynska-Walczak, A., Bialkowski, S., Eds.; CumInCAD Papers: Lodz, Poland, 2018; Volume 1, pp. 791–800.
5. Perrot, A.; Rangeard, D.; Pierre, A. Structural built-up of cement-based materials used for 3D-printing extrusion techniques. *Mater. Struct.* **2016**, *49*, 1213–1220. https://doi.org/10.1617/s11527-015-0571-0.
6. Le, T.T.; Austin, S.A.; Lim, S.; Buswell, R.A.; Law, R.; Gibb, A.G.F.; Thorpe, T. Hardened properties of high-performance printing concrete. *Cem. Concr. Res.* **2012**, *42*, 558–666. https://doi.org/10.1016/j.cemconres.2011.12.003.
7. Ferretti, E.; Moretti, M.; Chiusoli, A.; Naldoni, L.; De Fabritiis, F.; Visonà, M. Mechanical Properties of a 3D-Printed Wall Segment Made with an Earthen Mixture. *Materials* **2022**, *15*, 438. https://doi.org/10.3390/ma15020438.
8. Perrot, A.; Rangeard, D.; Courteille, E. 3D printing of earth-based materials: Processing aspects. *Constr. Build. Mater.* **2018**, *172*, 670–676. https://doi.org/10.1016/j.conbuildmat.2018.04.017.
9. Gomaa, M.; Jabi, W.; Veliz Reyes, A.; Soebarto, V. 3D printing system for earth-based construction: Case study of cob. *Autom. Constr.* **2021**, *124*, 103577. https://doi.org/10.1016/j.autcon.2021.103577.
10. Etzion, Y.; Saller, M. Earth Construction—A Review of Needs and Methods. *Arch. Sci. Rev.* **1987**, *30*, 43–48. https://doi.org/10.1080/00038628.1987.9697284.
11. Olivier, M.; Mesbah, A. Influence of different parameters on the resistance of earth, used as a building material. In Proceedings of the International Conference on Mud Architecture, Trivandrum, India, 25–27 November 1987.
12. Germen, A. The endurance of earths as building material and the discreet but continuous charm of adobe. *METU J. Fac. Archit.* **1979**, *5*, 37–68.
13. Keefe, L. *Earth Building Methods and Materials, Repair and Conservation*; Taylor & Francis: New York, NY, USA, 2005.
14. Deboucha, S.; Hashim, R. A review on bricks and stabilized compressed earth blocks. *Sci. Res. Essays* **2011**, *6*, 499–506.
15. Treloar, G.; Owen, C.M.; Fay, M.R. Enviromental assessment of rammed earth construction systems. *Struct. Surv.* **2001**, *19*, 99–105.
16. Pacheco-Torgal, F.; Jalali, S. Earth construction: Lessons from the past for future eco-efficient construction. *Constr. Build. Mater.* **2012**, *29*, 512–519. https://doi.org/10.1016/j.conbuildmat.2011.10.054.
17. Gallipoli, D.; Bruno, A.W.; Perlot, C.; Salmon, N. *Raw Earth Construction: Is there a Role for Unsaturated Soil Mechanics?*; Taylor & Francis Group: London, UK, 2014.
18. Schroeder, H. *Sustainable Building with Earth*; Springer International Publishing: New York, NY, USA, 2016.
19. Walker, P. Editorial. *Proc. Inst. Civ. Eng. Constr. Mater.* **2016**, *169*, 239–240. http://dx.doi.org/10.1680/jcoma.2016.169.5.239.
20. Olivier, M.; Mesbah, A. Le matériau terre: Essai de compactage statique pour la fabrication de briques de terre compressées. *Bull. Liaison Lab. Ponts Chaussées* **1986**, *146*, 37–43.
21. Houben, H.; Guillaud, H. *Earth Construction: A Comprehensive Guide*; Intermediate Technology Publications: London, UK, 1994.
22. Rigassi, V. CRATerre-EAG. In *Compressed Earth Blocks: Manual of Production*; Friedrich Vieweg & Sohn: Braunschweig, Germany, 1995.
23. Walker, P. Strength, durability and shrinkage characteristics of cement stabilised soil blocks. *Cem. Concr. Compos.* **1995**, *17*, 301–310. https://doi.org/10.1016/0958-9465(95)00019-9.
24. Maniatidis, V.; Walker, P. *A Review of Rammed Earth Construction;* DTi Partners in Innovation Project 'Developing Rammed Earth for UK Housing', University of Bath: Bath, UK, 1 May 2003. Available online: https://people.bath.ac.uk/abspw/rammedearth/review.pdf (accessed on 20 December 2021).
25. Morel, J.-C.; Pkla, A.; Walker, P. Compressive strength testing of compressed earth blocks. *Constr. Build. Mater.* **2007**, *21*, 303–309. https://doi.org/10.1016/j.conbuildmat.2005.08.021.
26. Kouakou, C.H.; Morel, J.-C. Strength and elasto-plastic properties of non-industrial building materials manufactured with clay as a natural binder. *Appl. Clay Sci.* **2009**, *44*, 27–34. https://doi.org/10.1016/j.clay.2008.12.019.
27. Reddy, B.; Kumar, P.P. Cement stabilised rammed earth. Part A: Compaction characteristics and physical properties of compacted cement stabilised soils. *Mater. Struct.* **2011**, *4*, 681–693.
28. Reddy, B.; Kumar, P.P. Cement stabilised rammed earth. Part B: Compressive strength and stress–strain characteristics. *Mater. Struct.* **2011**, *44*, 695–707. https://doi.org/10.1617/s11527-010-9659-8.
29. Bruno, A.W.; Gallipoli, D.; Perlot, C.; Mendes, J. Effect of very high compaction pressures on the physical and mechanical properties of earthen materials. In Proceedings of the 3rd European Conference on Unsaturated Soils, Paris, France, 12–14 September 2016; Delage, P., Cui, Y.-J., Ghabezloo, S., Pereira, J.-M., Tang, A.-M., Eds.; Volume 9, p. 14004.
30. McGregor, F.; Heath, A.; Fodde, E.; Shea, A. Conditions affecting the moisture buffering measurement performed on compressed earth blocks. *Build. Environ.* **2014**, *75*, 11–18.
31. Fabbri, A.; Morel, J.-C.; Gallipoli, D. Assessing the performance of earth building materials: A review of recent developments. *RILEM Tech. Lett.* **2018**, *3*, 46–58. https://doi.org/10.21809/rilemtechlett.2018.71.
32. Verma, P.L.; Mehra, S.R. Use of soil-cement in house construction in the Punjab. *Indian Concr. J.* **1950**, *24*, 91–96.

33. Walker, P. *HB 195: The Australian Earth Building Handbook*; Standards Australia: Sydney, Australia, 2002.
34. Walker, P.; Keable, R.; Martin, J.; Maniatidis, V. *Rammed Earth: Design and Construction Guidelines*; IHS BRE: BRE Bookshop: Watford, UK, 2005.
35. Fitzmaurice, R. *Manual on Stabilized Soil Construction for Housing*; Technical Assistance Programme; United Nations: New York, NY, USA, 1958.
36. Spence, R.J.S. Predicting the performance of soil-cement as a building material in tropical countries. *Build. Sci.* **1975**, *10*, 155–159. https://doi.org/10.1016/0007-3628(75)90031-6.
37. Alley, P.J. Rammed Earth Construction. *N. Z. Eng.* **1948**, *3*, 582.
38. McHenry, P.G. *Adobe and Rammed Earth Buildings: Design and Construction*; The University of Arizona Press: Tucson, AZ, USA, 1984.
39. Koutous, A.; Hilali, El M. A Proposed Experimental Method for the Preparation of Rammed Earth Material. *Int. J. Eng. Tech. Res. (IJERT)* **2019**, *8*, 345–354. http://dx.doi.org/10.17577/IJERTV8IS070140.
40. Smith, E.W.; Austin, G.S. *Adobe, Pressed-Earth, and Rammed-Earth Industries in New Mexico*; Bulletin 127; New Mexico Bureau of Mines & Mineral Resources: Socorro, NM, USA, 1989.
41. Norton, J. *Building with Earth. A Handbook*, 2nd ed.; Intermediate Technology Publications: London, UK, 1997.
42. Gooding, D. *Soil Testing for Soil-Cement Block Preparation*; DTU Working Paper: WP38; University of Warwick: Coventry, UK, 1993.
43. Montgomery, D. *Physical Characteristics of Soils that Encourage SSB Breakdown during Moisture Attack*; Stabilised Soil Research Progress Report SSRPR03; University of Warwick: Coventry, UK, 1998.
44. VITA (Volunteers in Technical. Assistance). *Making Buildings Blocks with the CINVA-Ram Block Press, Volunteers in Technical Assistance*, 3rd ed.; Intermediate Technology Publications: London, UK, 1975.
45. Reddy, B.; Jagadish, K.S. Influence of soil composition on the strength and durability of soil-cement blocks. *Indian Concr. J.* **1995**, *69*, 517–524.
46. Walker, P.; Stace, T. Properties of some cement stabilised compressed earth blocks and mortars. *Mater. Struct.* **1997**, *30*, 545–551. https://doi.org/10.1007/bf02486398.
47. Reddy, B.; Lal, R.; Rao, K.N. Optimum Soil Grading for the Soil-Cement Blocks. *J. Mater. Civ. Eng.* **2007**, *19*, 139–148. https://doi.org/10.1061/(asce)0899-1561(2007)19:2(139).
48. Reddy, B.; Latha, M.S. Influence of soil grading on the characteristics of cement stabilised soil compacts. *Mater. Struct.* **2014**, *47*, 1633–1645. https://doi.org/10.1617/s11527-013-0142-1.
49. Burroughs, S. Soil Property Criteria for Rammed Earth Stabilization. *J. Mater. Civ. Eng.* **2008**, *20*, 264–273. https://doi.org/10.1061/(asce)0899-1561(2008)20:3(264).
50. Burroughs, S. Recommendations for the Selection, Stabilization, and Compaction of Soil for Rammed Earth Wall Construction. *J. Green Build.* **2010**, *5*, 101–114.
51. Reddy, B.; Kumar, P.P. Role of clay content and moisture on characteristics of cement stabilised rammed earth. In Proceedings of the 11th International Conference on Non-Conventional Materials and Technologies (NOCMAT), Bath University, Bath, UK, 6–9 September 2009.
52. Nagaraj, H.B.; Rajesh, A.; Sravan, M.V. Influence of soil gradation, proportion and combination of admixtures on the properties and durability of CSEBs. *Constr. Build. Mater.* **2016**, *110*, 135–144.
53. Bryan, A.J. Criteria for the suitability of soil for cement stabilization. *Build. Environ.* **1988**, *23*, 309–319. https://doi.org/10.1016/0360-1323(88)90037-6.
54. Reddy, B.; Jagadish, K.S. Properties of soil–cement block masonry. *Mason. Int.* **1989**, *3*, 80–84.
55. Ciancio, D.; Boulter, M. Stabilised rammed earth: A case study in Western Australia. *Proc. Inst. Civ. Eng. Eng. Sustain.* **2012**, *165*, 141–154. https://doi.org/10.1680/ensu.10.00003.
56. Heathcote, K.A. Durability of earthwall buildings. *Constr. Build. Mater.* **1995**, *9*, 185–189. https://doi.org/10.1016/0950-0618(95)00035-e.
57. Morel, J.-C.; Bui, Q.B.; Hamard, E. Weathering and durability of earthen material and structures. In *Modern Earth Buildings: Materials, Engineering, Constructions and Applications*; Hall, M.R., Lindsay, R., Krayenhoff, M., Eds.; Woodhead Publishing: Sawston (Cambridge), UK, 2012; pp. 282–303.
58. Guettala, A.; Abibsi, A.; Houari, H. Durability study of stabilized earth concrete under both laboratory and climatic conditions exposure. *Constr. Build. Mater.* **2006**, *20*, 119–127.
59. Bui, Q.B.; Morel, J.-C.; Venkatarama Reddy, B.V.; Ghayad, W. Durability of rammed earth walls exposed for 20 years to natural weathering. *Build. Environ.* **2009**, *44*, 912–919.
60. Beckett, C.; Ciancio, D. Durability of cement-stabilised rammed earth: A case study in Western Australia. *Aust. J. Civ. Eng.* **2016**, *14*, 54–62. https://doi.org/10.1080/14488353.2015.1092671.
61. Bruno, A.W.; Gallipoli, D.; Perlot, C.; Mendes, J. Effect of stabilisation on mechanical properties, moisture buffering and water durability of hypercompacted earth. *Constr. Build. Mater.* **2017**, *149*, 733–740. https://doi.org/10.1016/j.conbuildmat.2017.05.182.
62. Allinson, D.; Hall, M. Humidity buffering using stabilised rammed earth materials. *Proc. Inst. Civ. Eng. Constr. Mater.* **2012**, *165*, 335–344. https://doi.org/10.1680/coma.11.00023.
63. McGregor, F.; Heath, A.; Shea, A.; Lawrence, M. The moisture buffering capacity of unfired clay masonry. *Build. Environ.* **2014**, *82*, 599–607. https://doi.org/10.1016/j.buildenv.2014.09.027.

64. Oudhof, N.; Labat, M.; Magniont, C.; Nicot, P. 2015. Measurement of the hygrothermal properties of straw-clay mixtures. In Proceedings of the First International Conference on Bio-bas$ed Building Materials, Clermont Ferrand, France, 22–24 June 2015; Amziane, S., Sonebi, M., Eds.; RILEM Publications—Curran Associates, Inc.: New York, NY, USA, 2017; pp. 474–479.
65. Arrigoni, A.; Grillet, A.C.; Pelosato, R.; Dotelli, G.; Beckett, C.; Woloszyn, M.; Ciancio, D. Reduction of rammed earth's hygroscopic performance under stabilisation: An experimental investigation. *Build. Environ.* **2017**, *115*, 358–367.
66. Muguda Viswanath, S. Biopolymer Stabilised Earthen Construction Materials. Ph.D. Thesis, Durham University, Durham, UK, 2019.
67. Webb, D. Stabilised soil and the built environment. *Renew. Energy* **1994**, *5*, 1066–1080. https://doi.org/10.1016/0960-1481(94)90134-1.
68. Walker, P. Editorial. *Proc. Inst. Civ. Eng. Constr. Mater.* **2017**, *170*, 1–2. http://dx.doi.org/10.1680/jcoma.2017.170.1.1.
69. Readle, D.; Coghlan, S.; Smith, J.C.; Corbin, A.; Augarde, C.E. Fibre reinforcement in earthen construction materials. *Proc. Inst. Civ. Eng. Constr. Mater.* **2016**, *169*, 252–260. https://doi.org/10.1680/jcoma.15.00039.
70. Plank, J. Applications of biopolymers and other biotechnological products in building materials. *Appl. Microbiol. Biotechnol.* **2004**, *66*, 1–9. https://doi.org/10.1007/s00253-004-1714-3.
71. Yang, F.; Zhang, B.; Ma, Q. Study of sticky rice− lime mortar technology for the restoration of historical masonry construction. *Acc. Chem. Res.* **2010**, *43*, 936–944.
72. Maskell, D.; Heath, A.; Walker, P. Comparing the Environmental Impact of Stabilisers for Unfired Earth Construction. *Key Eng. Mater.* **2014**, *600*, 132–143. https://doi.org/10.4028/www.scientific.net/kem.600.132.
73. Reddy, B.; Kumar, P.P. Embodied energy in cement stabilised rammed earth walls. *Energy Build.* **2010**, *42*, 380–385. https://doi.org/10.1016/j.enbuild.2009.10.005.
74. Gallipoli, D.; Bruno, A.W.; Perlot, C.; Mendes, J. A geotechnical perspective of raw earth building. *Acta Geotech.* **2017**, *12*, 463–478. https://doi.org/10.1007/s11440-016-0521-1.
75. Lax, C. Life Cycle Assessment of Rammed Earth. Master's Thesis, University of Bath, Bath, UK, 2010.
76. Fujita, Y.; Ferris, F.G.; Lawson, R.D.; Colwell, F.S.; Smith, R.W. Subscribed content calcium carbonate precipitation by ureolytic subsurface bacteria. *Geomicrobiol. J.* **2000**, *17*, 305–318.
77. Renforth, P.; Manning, D.A.C.; Lopez-Capel, E. Carbonate precipitation in artificial soils as a sink for atmospheric carbon dioxide. *Appl. Geochem.* **2009**, *24*, 1757–1764. https://doi.org/10.1016/j.apgeochem.2009.05.005.
78. Ivanov, V.; Stabnikov, V. *Construction Biotechnology: Biogeochemistry, Microbiology and Biotechnology of Construction Materials and Processes*; Springer: Singapore, 2016.
79. Cabalar, A.F.; Canakci, H. Direct shear tests on sand treated with xanthan gum. *Proc. Inst. Civ. Eng. Ground Improv.* **2011**, *164*, 57–64. https://doi.org/10.1680/grim.800041.
80. Chen, R.; Zhang, L.; Budhu, M. Biopolymer Stabilization of Mine Tailings. *J. Geotech. Geoenvironmental Eng.* **2013**, *139*, 130128212232006. https://doi.org/10.1061/(asce)gt.1943-5606.0000902.
81. Chang, I.; Im, J.; Prasidhi, A.K.; Cho, G.-C. Effects of Xanthan gum biopolymer on soil strengthening. *Constr. Build. Mater.* **2015**, *74*, 65–72. https://doi.org/10.1016/j.conbuildmat.2014.10.026.
82. Chang, I.; Prasidhi, A.K.; Im, J.; Cho, G.-C. Soil strengthening using thermo-gelation biopolymers. *Constr. Build. Mater.* **2015**, *77*, 430–438. https://doi.org/10.1016/j.conbuildmat.2014.12.116.
83. Aguilar, R.; Nakamatsu, J.; Ramírez, E.; Elgegren, M.; Ayarza, J.; Kim, S.; Pando, M.A.; Ortega-San-Martin, L. The potential use of chitosan as a biopolymer additive for enhanced mechanical properties and water resistance of earthen construction. *Constr. Build. Mater.* **2016**, *114*, 625–637. https://doi.org/10.1016/j.conbuildmat.2016.03.218.
84. Ayeldeen, M.K.; Negm, A.M.; El Sawwaf, M.A. Evaluating the physical characteristics of biopolymer/soil mixtures. *Arab. J. Geosci.* **2016**, *9*, 329–339. https://doi.org/10.1007/s12517-016-2366-1.
85. Pacheco-Torgal, F.; Ivanov, V.; Karak, N.; Jonkers, H. *Biopolymers and Biotech Admixtures for Eco-Efficient Construction Materials*; Woodhead Publishing: Sawston, UK, 2016.
86. Latifi, N.; Horpibulsuk, S.; Meehan, C.L.; Abd Majid, M.Z.; Tahir, M.M.; Mohamad, E.T. Improvement of Problematic Soils with Biopolymer—An Environmentally Friendly Soil Stabilizer. *J. Mater. Civ. Eng.* **2017**, *29*, 04016204.
87. Nakamatsu, J.; Kim, S.; Ayarza, J.; Ramírez, E.; Elgegren, M.; Aguilar, R. Eco-friendly modification of earthen construction with carrageenan: Water durability and mechanical assessment. *Constr. Build. Mater.* **2017**, *139*, 193–202. https://doi.org/10.1016/j.conbuildmat.2017.02.062.
88. Qureshi, M.U.; Chang, I.; Al-Sadarani, K. Strength and durability characteristics of biopolymer-treated desert sand. *Géoméch. Eng.* **2017**, *12*, 785–801. https://doi.org/10.12989/gae.2017.12.5.785.
89. Chen, C.; Wu, L.; Perdjon, M.; Huang, X.; Peng, Y. The drying effect on xanthan gum biopolymer treated sandy soil shear strength. *Constr. Build. Mater.* **2019**, *197*, 271–279. https://doi.org/10.1016/j.conbuildmat.2018.11.120.
90. Chang, I.; Im, J.; Cho, G.-C. Introduction of Microbial Biopolymers in Soil Treatment for Future Environmentally-Friendly and Sustainable Geotechnical Engineering. *Sustainability* **2016**, *8*, 251. https://doi.org/10.3390/su8030251.
91. Stabnikov, V.; Ivanov, V.; Chu, J. Construction Biotechnology: A new area of biotechnological research and applications. *World J. Microbiol. Biotechnol.* **2015**, *31*, 1303–1314. https://doi.org/10.1007/s11274-015-1881-7.
92. Komnitsas, K.; Zaharaki, D. Geopolymerisation: A review and prospects for the minerals industry. *Miner. Eng.* **2007**, *20*, 1261–1277. https://doi.org/10.1016/j.mineng.2007.07.011.

93. De Silva, P.; Sagoe-Crenstil, K.; Sirivivatnanon, V. Kinetics of geopolymerization: Role of Al2O3 and SiO2. *Cem. Concr. Res.* **2007**, *37*, 512–518. https://doi.org/10.1016/j.cemconres.2007.01.003.
94. Fletcher, R.A.; MacKenzie, K.J.; Nicholson, C.L.; Shimada, S. The composition range of aluminosilicate geopolymers. *J. Eur. Ceram. Soc.* **2005**, *25*, 1471–1477. https://doi.org/10.1016/j.jeurceramsoc.2004.06.001.
95. Songpiriyakij, S.; Kubprasit, T.; Jaturapitakkul, C.; Chindaprasirt, P. Compressive strength and degree of reaction of bio-mass-and fly ash-based geopolymer. *Constr. Build. Mater.* **2010**, *24*, 236–240.
96. Cheng, T.-W.; Chiu, J. Fire-resistant geopolymer produced by granulated blast furnace slag. *Miner. Eng.* **2003**, *16*, 205–210. https://doi.org/10.1016/s0892-6875(03)00008-6.
97. Colombo, D. WASP arriva a 12 metri, 2015. Available online: https://www.01factory.it/wasp-arriva-a-12-metri (accessed on 20 December 2021).
98. Colombo, D. La BigDelta di WASP nel parco della stampa 3D, 2016. Available online: https://www.01factory.it/la-bigdelta-di-wasp-nel-parco-della-stampa-3d (accessed on 20 December 2021).
99. Colombo, D. Tecla, la casa stampata in 3D in terra cruda, 2021. Available online: https://www.01building.it/progetti/tecla-casa-stampata-3d-terra-cruda (accessed on 20 December 2021).
100. Singh, B. Rice husk ash. In *Waste and Supplementary Cementitious Materials in Concrete*; Elsevier: Amsterdam, The Netherlands, 2018; pp. 417–460.
101. Omatola, K.; Onojah, A. Rice Husk as a Potential Source of High Technology Raw Materials: A Review. *J. Phys. Sci. Innov.* **2012**, *4*, 1–6.
102. Mathur, A.; Singh, U.; Vijay, Y.; Hemlata, M.S.; Sharma, M. Analysing performance for generating power with renewable energy source using rice husk as an alternate fuel. *Control Theory Inform.* **2013**, *3*, 64–71.
103. Mohd Basri, M.S.; Mustapha, F.; Mazlan, N.; Ishak, M.R. Rice Husk Ash-based Geopolymer Binder: Compressive Strength, Optimize Composition, FTIR Spectroscopy, Microstructural and Potential as Fire Retardant Material. *Polymers* **2021**, *13*, 4373.
104. Moretti, M.; Chiusoli, A.; Naldoni, L.; De Fabritiis, F.; Visonà, M. Earthen 3d printed constructions towards a new high-efficient way of building. In *Past and Present of the Earthen Architectures in China and Italy*; Luvidi, L., Fratini, F., Rescic, S., Zhang, J., Eds.; Cnr Edizioni: Roma, Italy, 2021; pp. 147–155.
105. Cengiz, O.; Chen, Y.; Datta, I.; Du, Y.; Foroughi, A.; Kriki, P.; Liao, Y.F.; Loonawat, B.V.; Randeria, S.C.; Salahinejhad, P.; et al. *Architecture of Continuity: From Materiality to Environment—Open Thesis Fabrication 2018–2019*; Iaac: Barcelona, Spain, 2019.
106. Walker, P. Characteristics of Pressed Earth Blocks in Earth Blocks. In Proceedings of the 11th International Brick/Block Masonry Conference, Tonji University, Shanghai, China, 14–16 October 1997; pp. 14–16.
107. Aubert, J.E.; Maillard, P.; Morel, J.-C.; Al Rafii, M. Towards a simple compressive strength test for earth bricks? *Mater. Struct.* **2016**, *49*, 1641–1654. https://doi.org/10.1617/s11527-015-0601-y.
108. Ferretti, E.; Di Leo, A.; Viola, E. A novel approach for the identification of material elastic constants. In *CISM Courses and Lec-ture—sProblems in Structural Identification and Diagnostics: General Aspects and Applications, Proceedings of the Workshop on Problems in Structural Identification and Diagnostics, Bologna, Italy, 15–16 July 2002*; Davini, C., Viola, E., Eds.; Springer-Verlag: Vienna, Austria, 2003; Volume 471, pp. 117–131.
109. Ferretti, E. Experimental procedure for verifying strain-softening in concrete. *Int. J. Fract.* **2004**, *126*, L27–L34. https://doi.org/10.1023/b:frac.0000026384.55711.4a.
110. Ferretti, E. On nonlocality and locality: Differential and discrete formulations. In Proceedings of the 11th International Confer-ence on Fracture 2005, Turin, Italy, 20–25 March 2005; International Congress on Fracture (ICF)—Curran Associates, Inc.: New York, NY, USA, 2010; Volume 3, pp. 1728–1733.
111. Ferretti, E.; Di Leo, A. Cracking and creep role in displacements at constant load: Concrete solids in compression. *CMC-Comput. Mater. Continua.* **2008**, *7*, 59–79.
112. Ferretti, E. Shape-effect in the effective laws of plain and rubberized concrete. *CMC-Comput. Mater. Continua.* **2012**, *30*, 237–284.
113. Ferretti, E. A discussion of strain-softening in concrete. *Int. J. Fract.* **2004**, *126*, L3–L10. https://doi.org/10.1023/b:frac.0000025302.13043.4a.
114. Ferretti, E. On Strain-softening in Dynamics. *Int. J. Fract.* **2004**, *126*, L75–L82. https://doi.org/10.1023/b:frac.0000031188.52201.de.
115. Ferretti, E. The Cell Method: An enriched description of physics starting from the algebraic formulation. *CMC-Comput. Mater. Continua* **2013**, *36*, 49–71.
116. Ferretti, E.; Di Leo, A.; Viola, E. Computational aspects and numerical simulations in the elastic constants identification. In *CISM Courses and Lectures Problems in Structural Identification and Diagnostics: General Aspects and Applications, Proceedings of the Workshop on Problems in Structural Identification and Diagnostics, Bologna, Italy, 15–16 July 2002*; Dav-ini, C., Viola, E., Eds.; Springer-Verlag: Vienna, Austria, 2003; Volume 471, pp. 133–147.
117. Saadeldin, R.; Siddiqua, S. Geotechnical characterization of a clay–cement mix. *Bull. Eng. Geol. Environ.* **2013**, *72*, 601–608.
118. Porbaha, A.; Shibuya, S.; Kishida, T. State of the art in deep mixing technology. *Ground. Improv.* **2000**, *4*, 91–110. https://doi.org/10.1680/grim.2000.4.3.91.
119. Horpibulsk, S.; Rachan, R.; Suddeepong, A.; Chinkulkijniwat, A. Strength Development in Cement Admixed Bangkok Clay: Laboratory and Field Investigations. *Soils Found.* **2011**, *51*, 239–251. https://doi.org/10.3208/sandf.51.239.
120. Bergado, D.T.; Anderson, L.R.; Miura, N.; Balasubramaniam, A.S. *Soft Ground Improvement in Lowland and Other Environments*; ASCE Press: New York, NY, USA, 1996.
